# Molecular characterization of a whirlin-like protein with biomineralization-related functions from the shell of *Mytilus coruscus*

**DOI:** 10.1371/journal.pone.0231414

**Published:** 2020-04-08

**Authors:** Yuting Jiang, Qi Sun, Meihua Fan, Xiaolin Zhang, Wang Shen, Huanzhi Xu, Zhi Liao

**Affiliations:** Laboratory of Marine Biological Source and Molecular Engineering, College of Marine Science, Zhejiang Ocean University, Zhoushan, Zhejiang, P.R. China; Dalian Ocean University, CHINA

## Abstract

Mollusc shells are produced from calcified skeletons and have excellent mechanical properties. Shell matrix proteins (SMPs) have important functions in shell formation. A 16.6 kDa whirlin-like protein (WLP) with a PDZ domain was identified in the shell of *Mytilus coruscus* as a novel SMP. In this study, the expression, function, and location of WLP were analysed. The WLP gene was highly expressed and specifically located in the adductor muscle and mantle. The expression of recombinant WLP (rWLP) was associated with morphological change, polymorphic change, binding ability, and crystallization rate inhibition of the calcium carbonate crystals *in vitro*. In addition, an anti-rWLP antibody was prepared, and the results from immunohistochemistry and immunofluorescence analyses revealed the specific location of the WLP in the mantle, adductor muscle, and myostracum layer of the shell, suggesting multiple functions for WLP in biomineralization, muscle-shell attachment, and muscle attraction. Furthermore, results from a pull-down analysis revealed 10 protein partners of WLP in the shell matrices and a possible network of interacting WLPs in the shell. In addition, in this study, one of the WLP partners, actin, was confirmed to have the ability to bind WLP. These results expand the understanding of the functions of PDZ-domain-containing proteins in biomineralization and provide clues for determining the mechanisms of myostracum formation and muscle-shell attachment.

## Introduction

Bivalves are a widely spread molluscan class with more than 10 000 species [1], and they are characterized by an ability to build shells of different sizes, forms and structures. Bivalve shells are very durable compared with inorganic geological forms and have been important to research in the fields of bioengineering and bionics for dozens of years [2, 3]. In nature, shells are formed by a biologically controlled process, viz., biomineralization, which results in a composite material that is composed of approximately 95% calcium carbonate (aragonite and calcite) and less than 5% organic components (shell matrices) [4]. Shells have excellent mechanical properties because of the assembly of different shell layers/microstructures and shell matrices, which consist primarily of shell matrix proteins (SMPs) [4, 5]. SMPs play important roles in the formation and mechanical properties of the shell, although the underlying mechanisms have not yet been revealed in detail [6]. Under the control of SMPs, calcium carbonate crystals are deposited and form nanostructures with different morphologies and polymorphs and assemble into a complete shell through biomineralization [6, 7]. Studies on the structure and function of SMPs will advance the understanding of the process of shell formation, as well as the molecular mechanism that lead to the excellent mechanical properties of shells.

*Mytilus coruscus* is a mussel with important economic value in the East China Sea. Its shell is composed of nacre, fibrous prism, and myostracum layers, and through transcriptome-proteome strategies, more than 60 SMPs have been identified in the shell of *M*. *coruscus* [8]. Among the shell proteome, 8 SMPs were found in the myostracum layer, including a novel whirlin-like protein (WLP, GenBank Accession: QGA67049.1) [8]. The myostracum is buried in the nacre layer of the shell and is exposed on the inner shell surface where the adductor muscle is attached, forming the adductor muscle scar (AMS) of each shell valve [8, 9]. The myostracum layer plays an important role in muscle-shell attachment, controlling shell closure. However, studies on the organic matrix and the structural roles of the myostracum layer are rare.

The structure of *M*. *coruscus* WLP is similar to that of PDZ (Postsynaptic density/Discs large/Zonula occludens) and LIM (Lin11/Isl-1/Mec-3) domain proteins (PDLIMs) and WLP in other species, with a sequence identity < 60%. The PDLIM family comprises proteins with a PDZ domain combined with at least one LIM domain and has been documented to participate in cytoskeleton organization, cell differentiation, and oncogenesis [10–13]. The LIM domain has been reported to have mineralization-related functions in human osteosarcoma cells [14, 15], implying a possible function for PDLIMs in biomineralization. On the other hand, whirlin is critical for retinal photoreceptor and vestibular and cochlear hair cell functions in humans [16, 17]. Three whirlin isoforms were identified previously, including the N-terminal short isoform, the C-terminal short isoform, and the long isoform [18, 19]. The relationship between whirlin and biomineralization has not been described to date. Both PDLIM and whirlin contain a PDZ domain, which are abundant protein-protein interaction modules found in various proteins with many cellular and biological functions [20]. PDZ-binding proteins, such as TAZ (transcriptional coactivator with the PDZ-binding motif), are known to play important roles in osteogenic differentiation and bone formation [21, 22], reminding the possible roles of the PDZ domain in biomineralization.

PDZ-domain-containing proteins (PDCPs) have been identified from the shell of various *Mollusca species*, including those of *Mytilus* [8, 23], *Ostrea* [24], *Pinctada* [25], and *Perna* [26]. The roles of PDCPs in shell formation remain a mystery. Therefore, *M*. *coruscus* WLP was recombinantly expressed, and the functions of the recombinant proteins were investigated to explore their possible function and mechanism in shell formation. Furthermore, the location of the WLP in the mantle, adductor muscle and shell surface and the identification of the WLP protein partners were analysed in this study. Our findings provide clues and offer deep insight into the molecular mechanisms of the PDCPs associated with biomineralization.

## Materials and methods

### Ethics statement

All procedures were in accordance with the guidelines of the Regulations for the Administration of Laboratory Animals (Decree No. 2 of the State Science and Technology Commission of the People's Republic of China, November 14, 1988) and were approved by the Institutional Animal Care and Use Committee of Zhejiang Ocean University.

### Sequence analysis and the expression of the WLP gene in *M*. *coruscus* tissues

The full-length cDNA sequence of WLP was screened out from the transcriptomic data of *M*. *coruscus* mantle based on the LC-MS/MS data [8]. The cDNA sequence of WLP was confirmed using PCR with primers–ACCAACCCGTCTTCGTCCAACT- (forward primer) and–GTGCCAGCAACGTATTTAGACC- (revers primer), and further verified by PCR production sequencing. The WLP sequence was analysed using conventional bioinformatic tools, including ORF Finder, BLAST, MEGA 7, SignalP server, SMART domain prediction, Phyre secondary structure prediction, and SWISS-MODEL tertiary structure prediction tools.

Total RNA was extracted by TRIzol reagent from various tissues of six individual mussels, including the mantle, adductor muscle, gill, blood cell, and gonad. The first strand of cDNA was synthesized using the PrimeScript™ RT kit (Takara). Quantitative real-time PCR (qRT-PCR) analyses were performed with three independent replicates using SYBR® Premix Ex Taq™ (TaKaRa) on an MX3000P Real-Time PCR system (Stratagene, US). qRT-PCR was performed with specific primers derived from the WLP sequence, including WLP/F (TCCTTCCGTACAGTGGG) and WLP/R (CTGGTTTAGTTTGTGCTCC). The relative expression levels were measured using the 2^−ΔΔCt^ method [27] with β-actin (β-actin/F: 5'-ATGAAACCACCTACAACAGT-3'; β-actin/R: 5'-TAGACCCACCAATCCAGACG-3') as an internal reference. Statistical analysis of differences was performed by SPSS 16.0 with one-way ANOVA followed by Tukey's multiple range tests. Differences were considered significantly at P < 0.05.

### *In situ* hybridization of WLP

To determine the location of the WLP mRNA expressed in the mantle and adductor muscle, *in situ* hybridization was performed. The fixed tissues were dehydrated through an ethanol series and then subjected to a xylene bath prior to paraffin embedding. Paraffin blocks were sectioned to 5 μm thickness. After treatment with proteinase K at 37°C for 20 min, the sectioned tissues were washed with 0.1 M freshly prepared glycine solution for 1 min and PBS for 2 min. The tissues were immediately fixed in 4% paraformaldehyde for 10 min and then incubated at 65°C with a FAM-labelled probe (5’-FAM-CACGCCAUUACGUGAUAGCUUCUGAAUAUA-3’) for 48 h. The sectioned tissues were washed in formamide-4X SSC at 60°C. The signals for control samples were visualized with a substrate 4’,6-diamidino-2-phenylindole (DAPI) reagent and the positive signals were visualized by the excitation wavelength (492 nm) and the emission wavelength (518 nm).

### Recombinant expression and purification of WLP

The WLP gene was codon-optimized and synthesized for use in an *E*. *coli* expression system. *Nco I* and *Xho I* restriction sites were attached to the 5’ and 3’ ends of the optimized sequence, respectively. The synthetic codon-optimized genes were excised by *Nco I* and *Xho I* digestion and ligated into a pET/32α expression vector. The construct was designed to yield a recombinant protein product with a molecular weight (MW) of ~ 35 kDa that fused in-frame to a His_6_ tag, a Trx tag, and an enterokinase cleavage site.

The recombinant WLP (rWLP) was expressed in *E*. *coli* strain BL21 (DE3). *E*. *coli* cells containing *rWLP/pET/32α* were grown in Luria–Bertani (LB) liquid medium (Sangon Biotech, Shanghai, China) with 10 μg/mL ampicillin at 37°C. Isopropyl-D-thiogalactopyranoside (IPTG) at a final concentration of 1 mM was used for the induction of the rWLPs. The induced cells were grown for 4 h, harvested and centrifuged at 1000 ×g for 15 min at 4°C, and the pellets were stored at -20°C for later use.

The cell pellets were dissolved in ice-cold lysis buffer (10 mM imidazole, 50 mM PBS, 100 mM NaCl, and 1 M EDTA, pH 8.0) and homogenized using a sonicator at 4°C. The inclusion bodies were harvested by centrifugation (8000 ×g, 10 min, 4°C) and dissolved overnight in a buffer (10 mM imidazole, 8 M urea, 100 mM NaCl, and 100 mM PBS, pH 8.0) at 4°C. Using a Ni-NTA column (Sangon Biotech, Shanghai, China), the rWLP was purified by elution buffer (300 mM imidazole, 8 M urea, 100 mM NaCl, and 100 mM PBS, pH 8.0). Isolated rWLP was refolded into a buffer containing oxidized and reduced glutathione (GSH/GSSG) and dialysed with a urea concentration gradient (0 ~ 8 M) [28].

After being refolded into buffer, the rWLP was digested by enterokinase to remove the translated vector sequence in the N-terminus. The digestion was performed in a buffer (250 mM Tris-HCl, 500 mM NaCl, and 2 mM CaCl_2_, pH 7.6) with 2 IU enterokinase incubated for 16 h at room temperature. Digested rWLPs were isolated by high-performance liquid chromatography (HPLC, Waters 650E, USA) with a reverse-phase C4 column (4.6 mm×250 mm, 300 Å, Agilent). The eluted protein fraction from HPLC was lyophilized and stored at -20°C before use. SDS-PAGE was performed with a 12% polyacrylamide gel, and the protein bands were visualized using Coomassie Brilliant Blue R-250.

### Functional analysis of rWLP

*In vitro* crystal growth experiments [29] were performed to test the effects of rWLP on the morphology of calcite and aragonite crystals. The rWLP was incubated in a freshly prepared saturated solution of calcium carbonate [30] on a siliconized cover glass with or without magnesium chloride. The crystallization experiments were carried out with various concentrations of rWLP (10, 30, and 50 μg/mL). The morphology of the calcium carbonate crystals induced by rWLP was observed through a Nova Nano 450 (FEI) scanning electron microscope (SEM). The polymorphism of the calcium carbonate crystals was further identified by FTIR spectroscopy (Nicolet Nexus 670).

Crystal binding experiments were performed to test the interaction between rWLP and calcite or aragonite calcium carbonate. Calcite and aragonite calcium carbonate crystals were freshly prepared using the methods of Yan *et al* [30]. The rWLP was dissolved (1 mg/mL, as sample I) and incubated with calcite or aragonite crystals at room temperature for 1 h. After centrifugation (10 000 ×g, 15 min), the supernatants were collected as sample II. The sediments were decalcified with 5% acetic acid and centrifuged (10 000 ×g, 15 min), and the supernatants were dialysed (1 kDa cut off) and used as sample III. Samples I ~ III were analysed by SDS-PAGE.

The inhibition of the rWLP on calcium carbonate precipitation was tested using the methods provided by Liang *et al* [29] with minor modifications. Briefly, a 10 mM calcium chloride solution containing rWLP at various concentrations (10, 30, and 50 μg/mL) was dropped into a 96-well plate, and the plate was placed in a closed desiccator. Solid ammonium carbonate was added to the desiccator to initiate calcium carbonate precipitation. The turbidity of the calcium chloride solution was monitored every minute for 10 min by measuring the absorbance at 630 nm with a microplate reader (Synergy H1, BioTek).

### Polyclonal antibody preparation and immunohistochemistry analysis

The purified rWLP was enriched and submitted to HuaAn Biotechnology Co., Ltd. (Hangzhou, China) for polyclonal antibody production. Briefly, a polyclonal antibody was prepared by immunizing New Zealand rabbits with 0.5 mL of rWLP (1 mg/mL) and an equal volume of complete adjuvant. Three booster injections, each containing 0.5 mL of rWLP (1 mg/mL) plus incomplete adjuvant, were subsequently given at one-week intervals. The antiserum was collected through the carotid artery 7 d after the last immunization and further purified by a protein A/G column.

The specificity of the antibody was assayed by western blotting with acid soluble and acid insoluble matrices extracted from three layers (nacre, myostracum, and fibrous prism) of the *M*. *coruscus* shell. The shell matrices were extracted as described previously [8] and the proteins separated by SDS-PAGE. The PAGE gel was then transblotted onto a PVDF membrane. The anti-rWLP polyclonal antibody (1:2000) was used as the primary antibody, and a horseradish peroxidase-labelled goat anti-rabbit IgG (1:10,000; HuaAn Biotechnology Co., Ltd.) was used as the secondary antibody. The western blots were visualized using a 3,3’,5,5’-tetramethylbenzidine-stabilized substrate.

To determine the tissue distribution of natural WLPs, tissues (mantle and adductor muscle) of *M*. *coruscus* were collected and fixed overnight in 10% formaldehyde and then dehydrated through an ascending ethanol gradient. Sections (4 μm) were cut by a microtome and collected on coated slides for immunohistochemistry. After dewaxing and rehydration, the slides were incubated overnight with the anti-rWLP antibody (1:200) supplemented with 1% BSA at 37°C. The primary antibodies were detected using a peroxidase-conjugated antibody against rabbit IgG and stained with a DAB solution. The sections were examined and photographed using a microscope (DFC450C, Leica, Germany).

### His-tag affinity pull-down assay

The rWLP contains a His_6_ tag in its sequence, and Ni-NTA beads (Sangon, China) were used for binding the rWLP. After binding with rWLP, the Ni-NTA beads were washed with binding buffer (20 mM Tris-HCl, 150 mM NaCl, and 10 mM imidazole, pH 8.0) and then incubated with total proteins extracted from the shell of *M*. *coruscus* [8] for 4 h at 4°C and then washed with elution buffer (20 mM Tris-HCl, 300 mM NaCl, and 300 mM imidazole, pH 8.0). The eluted protein samples were analysed by LC-MS/MS after digestion with trypsin. The LC-MS/MS experiments were performed on a Q Exactive Plus MS coupled with Easy-nLC (Thermo Scientific). The MS data were analysed using MaxQuant software (version 1.6.1.0.), and searched against the mantle transcriptome database of *M*. *coruscus* (Accession: SRX792025) [8]. The database search results were filtered and exported with a <1% false discovery rate (FDR) at the peptide-spectrum-matched level and protein level.

### Biolayer interferometry

The binding of rWLP with actin was measured by Bio-Layer Interferometry (BLI) on an Octet RED BLI (Pall ForteBio) at 25°C [31]. The rWLP (1 mM) was dissolved in PBS buffer (pH 7.4) containing 0.05% (v/v) Tween 20 and 0.1% (v/v) BSA and then loaded onto an APS biosensor (Pall ForteBio) coated with actin. The procedure was as follows: 60 s for the baseline 1, 900 s for loading, 300 s for the baseline 2, 300 s for association, and 120 s for dissociation. The raw data were processed by subtraction and alignment, and the affinity constant (K_D_) was determined using ForteBio Data Analysis 10.0 software [32].

### Double-labelling immunofluorescence analysis

The location and the interaction of WLP with actin on the shell inner surface were determined by double-labelling immunofluorescence using two antibodies: rabbit anti-rWLP antibody (1:500, prepared for this study) and mouse anti-actin monoclonal antibody (1:500, Hangzhou HuaAn). Briefly, the shell was cut into pieces of ~1 cm^2^ containing the AMS region. The shell pieces were washed and sonicated in 5% NaOH to remove remaining adductor muscle and organic contaminants from the shell surface, and then, the sample was soaked in a stationary liquid containing 10% formaldehyde and 4% formic acid for 24 h. Then, the shell pieces were cleaned and treated with 0.25% Triton X-100 for 30 min and blocked with 10% negative goat serum for 1 h at 37°C. Immunostaining was performed by incubating the shell samples with anti-rWLP polyclonal antibody (1:500) and Alexa 488-conjugated goat-anti-rabbit antibody (1:5000), or mouse anti-actin monoclonal antibody and Alexa 555-conjugated goat-anti-mouse antibody (1:5000). The stained sections were examined with a fluorescence microscope (DMIL LED FLUO, Leica, Germany) equipped with a DFC450C digital imaging system (Leica, Germany). Deproteinized shell samples treated with 20% NaOH at 60°C for 1 h were used as negative controls.

## Results

### Features of the WLP sequence

**Fig 1:**
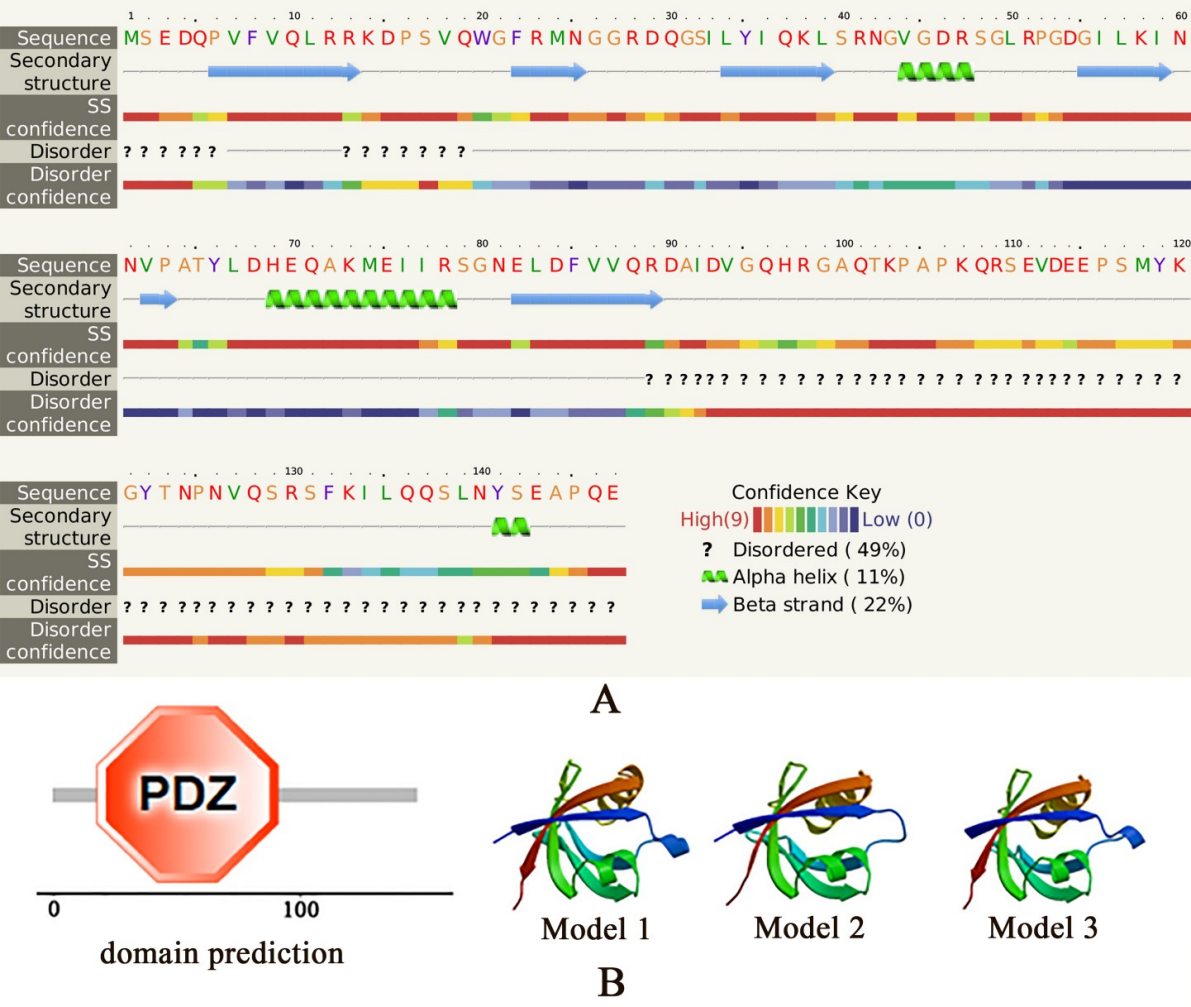
The structural features of WLP. A: the secondary structure of WLP predicted by Phyre. The regions adopting putative α-helix and β-sheet are represented as spiral and arrow, respectively. B: the domain prediction and spatial structure of WLP. Domain of WLP was predicted by SMART and one PDZ domain was showed. Spatial structure of WLP was predicted by SWISS-MODEL. Model 1~3 represent the predicted structures with highest score using 6qji.4.A, 6qji.6.A, and 6qji.1.A as templates, respectively.

The open reading frame encoding native WLP is 444 bp and encodes a 147 amino acid (AA) polypeptide (S1 Fig), with a theoretical molecular weight of 16.6 kDa and an isoelectric point (*pI*) of 7.97. One PDZ domain (residues 18 to 91) was detected in the WLP, and no signal peptide was detected (Fig 1). The secondary structure of the WLP is primarily composed of 11% α-helices and 23% β-sheets (Fig 1A), which form a predicted tertiary structure with characteristics similar to the standard conformation of the PDZ domain with five β-strands and two α-helices (Fig 1B). The protein BLAST results revealed that WLP shares sequence identity (50~60%) with PDLIM proteins and whirlin-like proteins of *Crassostrea virginica* and *Mizuhopecten yessoensis*, respectively (S1 Table). To analyse the evolutionary relationships of WLP gene, a rooted neighbor-joining phylogenetic tree was constructed using MEGA 7 software with bootstrap test of 1000 times. A total of 25 representative homologues were selected to build the phylogenetic tree (S2 Fig), in which five conspicuous branches were generated: PDLIM ZASP and whirlin-like in bivalves, PDLIM 2 in gastropods, PDLIM 1 in cephalopods, PDLIM 7 in brachiopods, and PDLIMs in flatworms. Remarkably, WLP of *M*. *coruscus* was more closely related to those in the same subfamily from bivalves than to the other PDLIM s from other species (S2 Fig), which implied a relatively high homology of WLP with PDLIMs and whirlin from bivalves.

### Tissue expression and *in situ* hybridization

**Fig 2:**
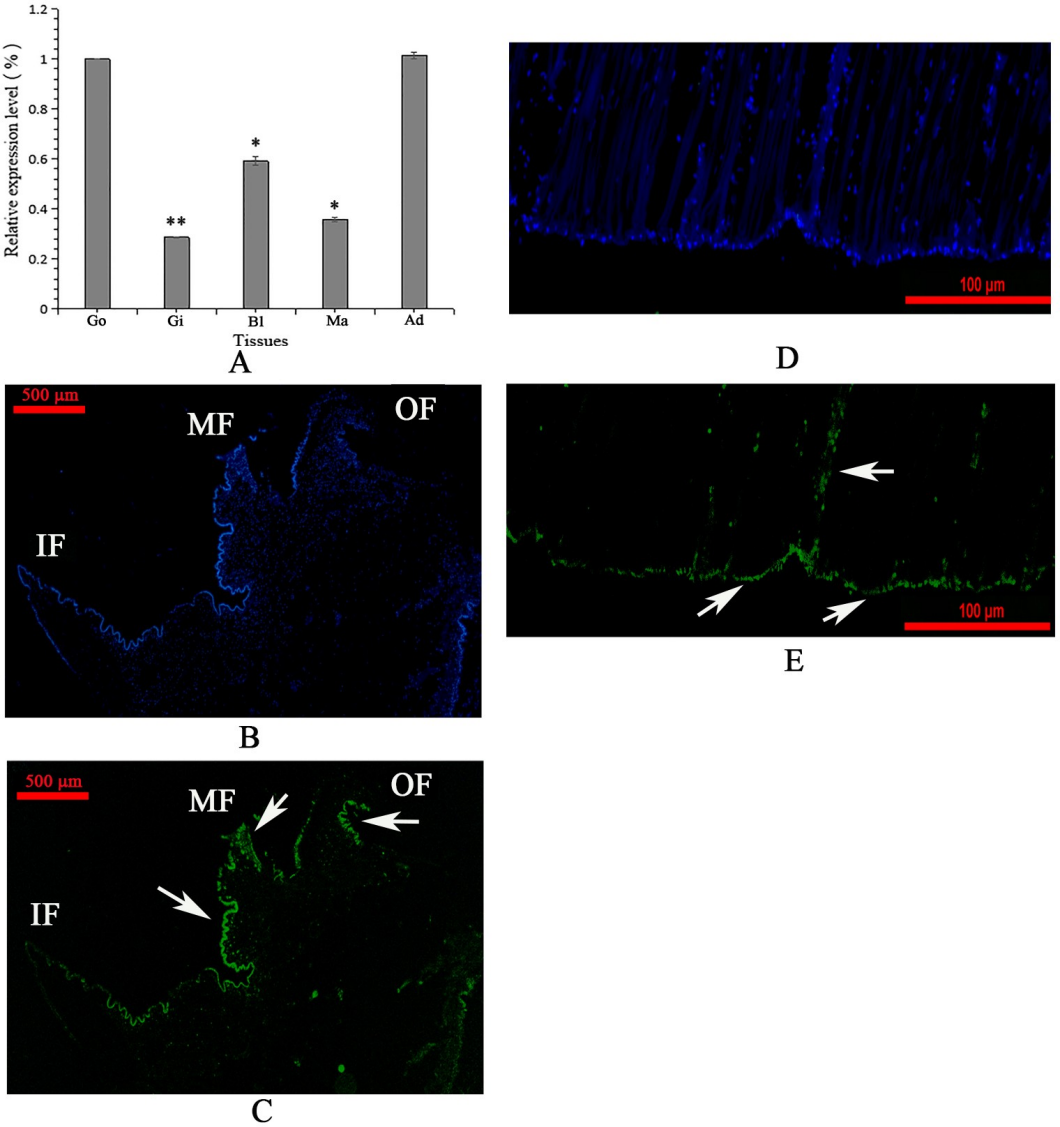
Tissue-specific expression (A) and *in situ* hybridization (B-E) of WLP gene. Go, gonad; Gi, gill; Bl, blood cell; Ma, mantle; Ad, adductor muscle. Values for qRT-PCR are means ± SD of three replicates. Significant difference relative to the expression level of adductor muscle was indicated with asterisk (*, *p*<0.05; **, *p*<0.01). B: control sample of *in situ* hybridization in the mantle; C: expression of WLP (green color, denoted by white arrows) in the mantle; D: control sample of *in situ* hybridization in the adductor muscle; E: expression of WLP (green color, denoted by white arrows) in the adductor muscle. The scale bar, 500 μm for B and C, and 100 μm for D and E.

The tissue-specific expression of WLP was investigated by qRT-PCR. The results of qRT-PCR showed that WLP gene was expressed in all tested tissues with high levels in the adductor muscle and gonad, and relatively low levels in blood cells, mantle, and gill (P<0.05) (Fig 2A). Using FAM-labelled WLP-specific probes, strong signals were detected at the edge of the middle fold and outer fold of the mantle and at the bottom of the adductor muscle near the shell surface (Fig 2B–2E).

### Expression and purification of recombinant WLP

**Fig 3:**
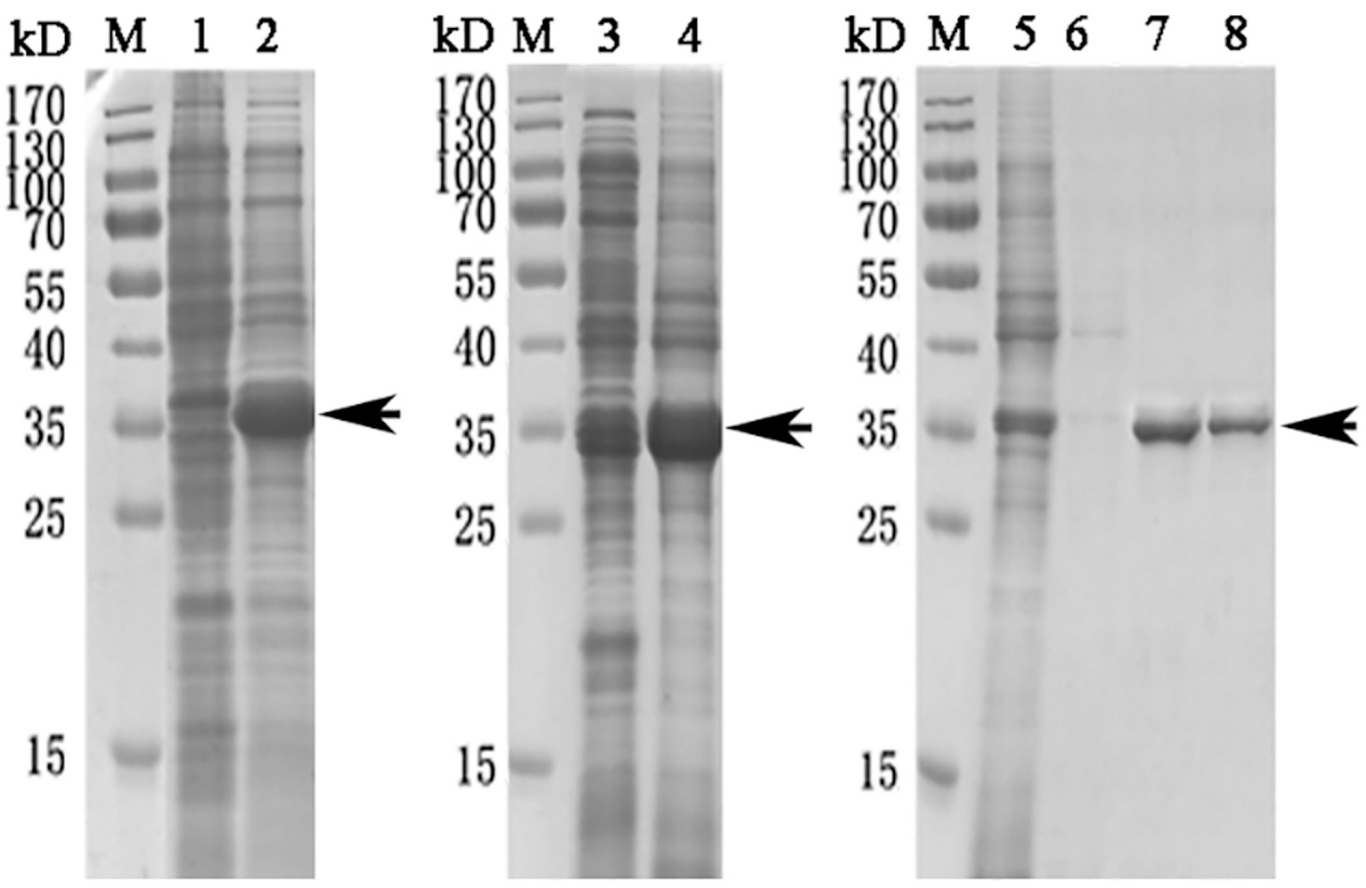
Expression and isolation of rWLP. Lane M, Protein Marker; Lane 1, negative control without the IPTG induction; Lane 2, expression of rWLP with the induction of IPTG; Lane 3, the supernatant of cell lysate; Lane 4 and 5, the debris of cell lysate; Lane 6, eluted rWLP from Ni-NTA column with 30 mM imidazole. Lane 7, eluted rWLP from Ni-NTA column with 300 mM imidazole; Lane 8, eluted rWLP from Ni-NTA column with 500 mM imidazole. The protein band with ~35 kDa (indicated by an arrow) corresponds to the rWLP.

**Fig 4:**
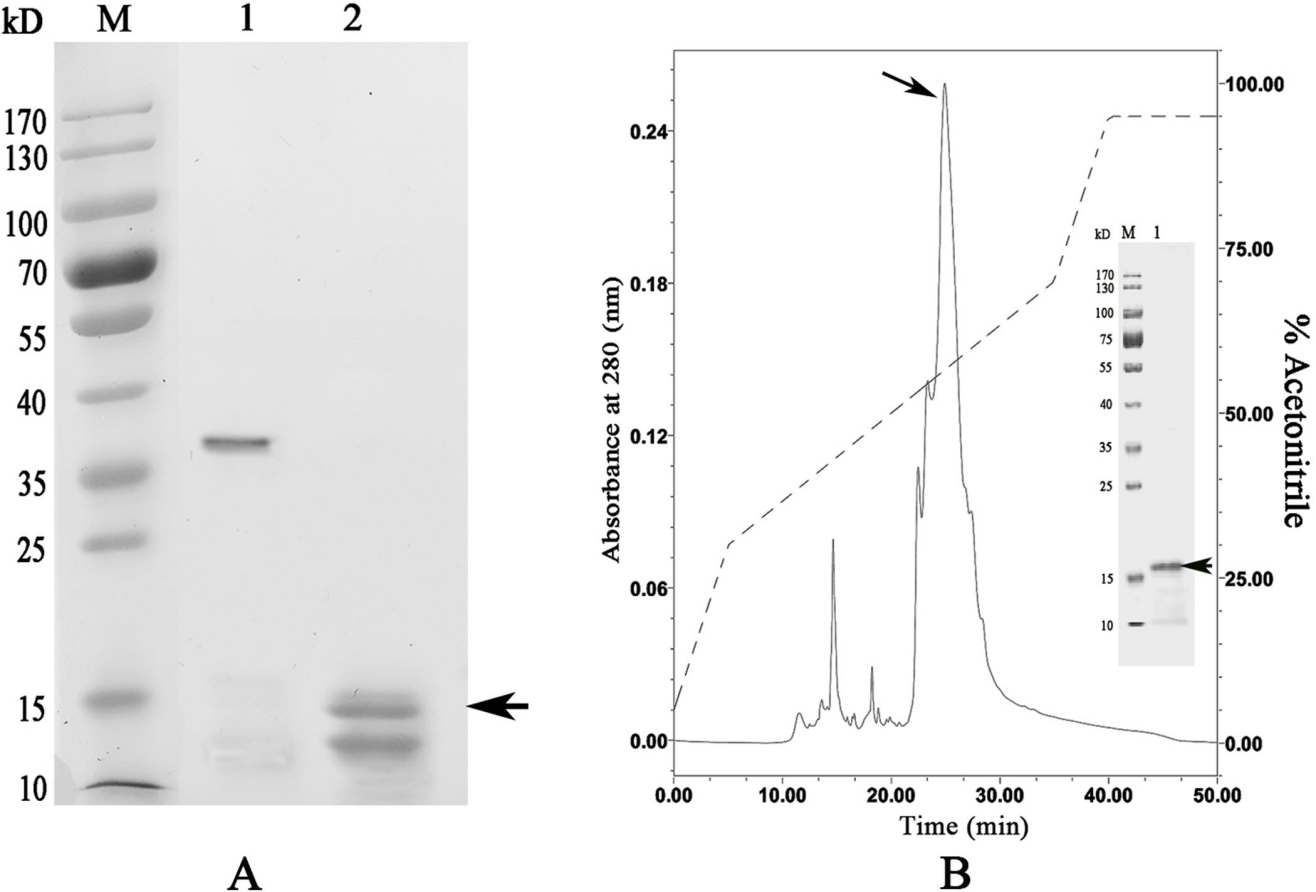
Enterokinase digestion (A) and HPLC purification of rWLP after digestion (B). Lane M, Protein Marker; lane 1, rWLP before enterokinase digestion; lane 2, rWLP after enterokinase digestion. The protein band with ~16 kDa (indicated by an arrow) corresponds to the rWLP.

The codon-optimized WLP gene was successfully expressed by *E*. *coli* upon induction with IPTG. The results from the SDS-PAGE experiment revealed predominant expression of the rWLP in inclusion bodies with an expected theoretical MW of ~35 kDa (Fig 3). rWLP was isolated through a Ni-NTA column, refolded in buffer and then digested by enterokinase to remove the N-terminal tags. As shown in Fig 4, the MW of rWLP without N-terminal tags was ~16 kDa, which is close to the MW of native WLP, indicating that the tags were successfully removed. Using HPLC, the digested rWLP was further purified, and high purity was achieved (Fig 4).

### Functions of rWLP

**Fig 5:**
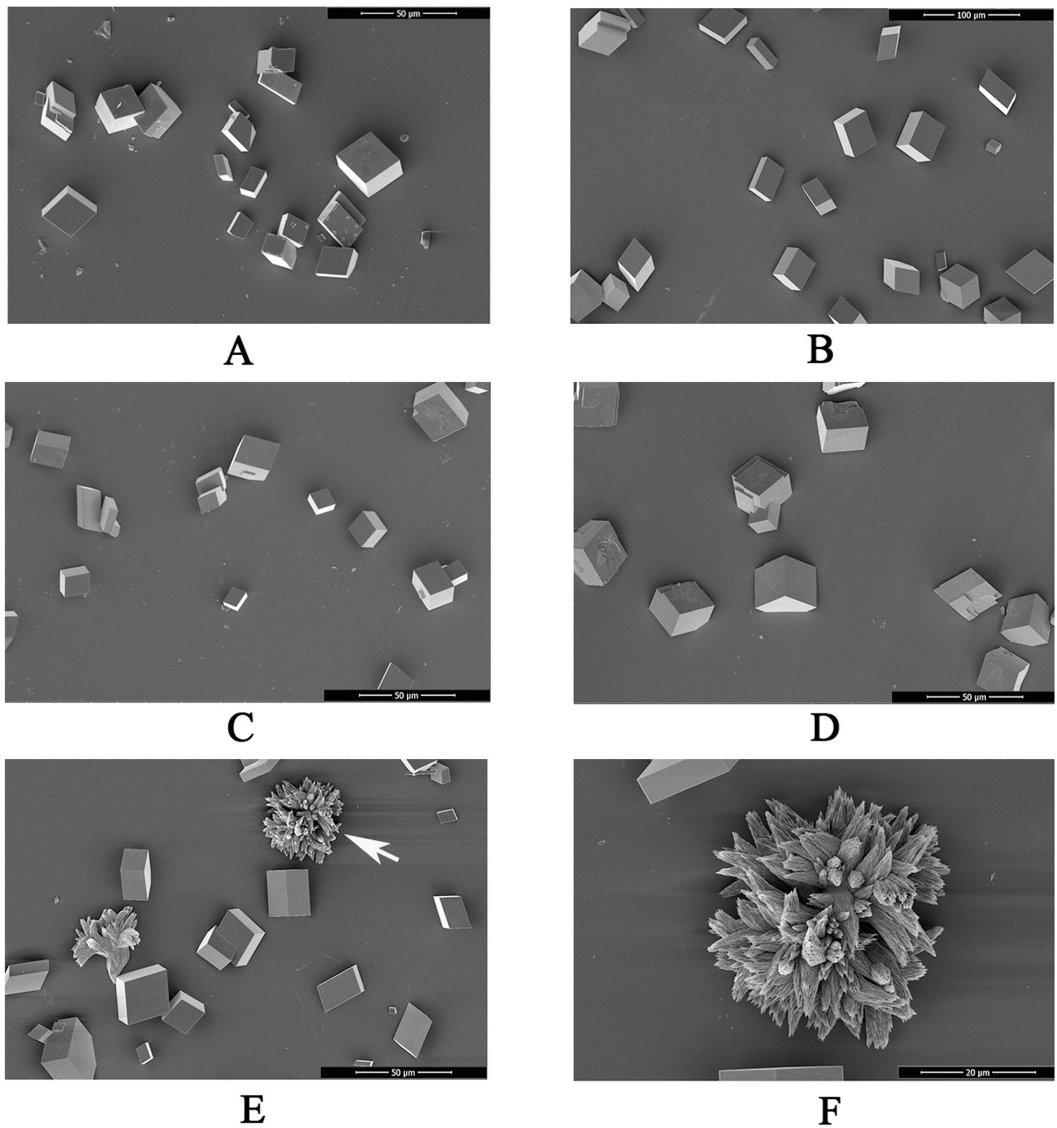
SEM images of *in vitro* calcite crystallization in the presence of rWLP at increasing concentrations. A: calcite crystals grown without protein induction. B: calcite crystals grown with 50 μg/mL BSA. C: calcite crystals grown with 10 μg/mL rWLP. D: calcite crystals grown with 30 μg/mL rWLP. E: crystals grown with 50 μg/mL rWLP. F: enlarged image of E.

**Fig 6:**
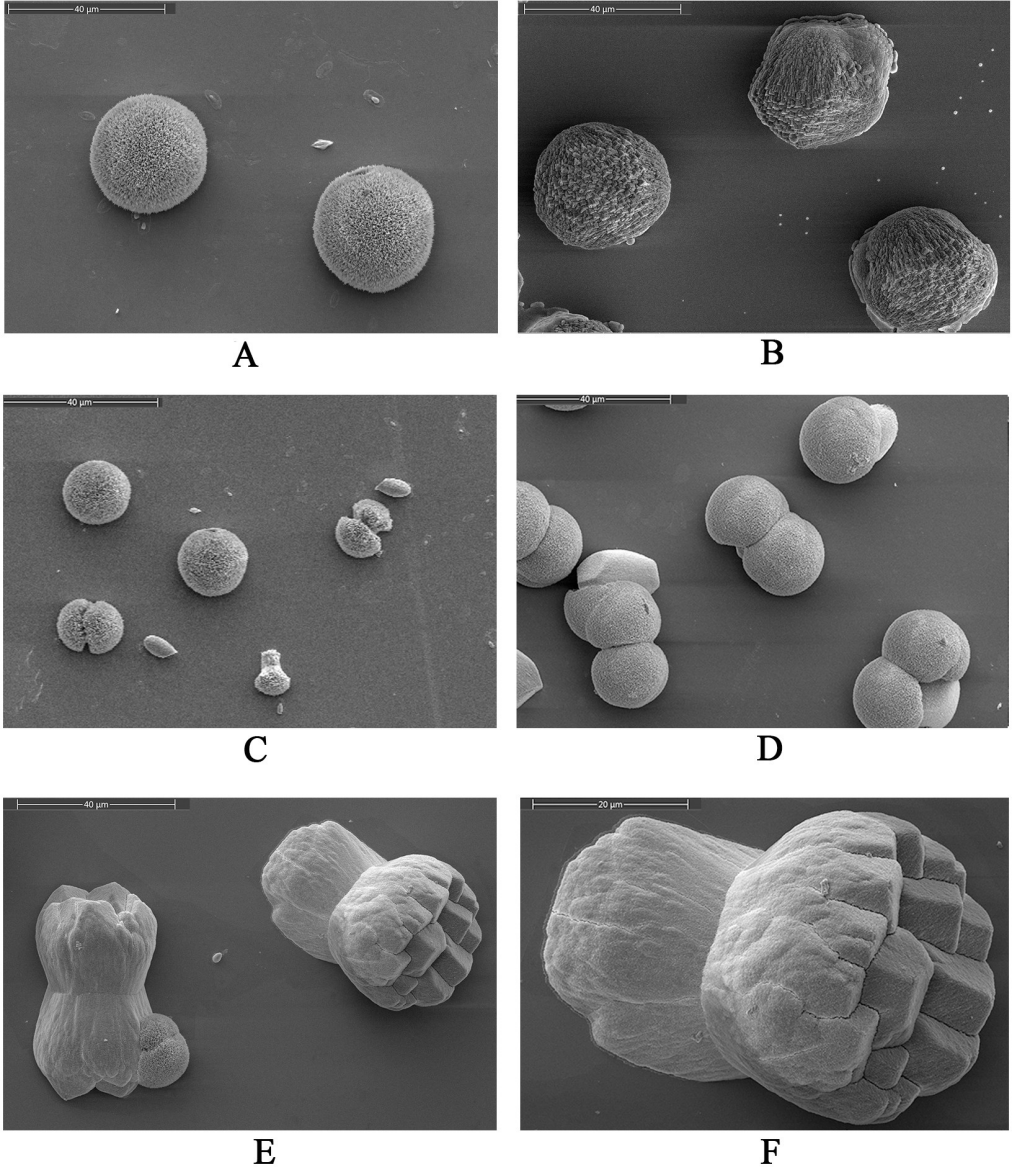
SEM images of *in vitro* aragonite crystallization in the presence of rWLP at increasing concentrations. A: aragonite crystals without protein induction. B: aragonite crystals grown with 50 μg/mL BSA. C: aragonite crystals grown with 10 μg/mL rWLP. D: aragonite crystals grown with 30 μg/mL rWLP. E: aragonite crystals grown with 50 μg/mL rWLP. F: enlarged image of E.

An *in vitro* calcium carbonate crystallization assay was performed, and the results revealed the influences of rWLP on the morphology of both calcite and aragonite crystals (Figs 5 and 6). For calcite not induced with protein (Fig 5A) or induced with 50 μg/mL BSA (Fig 5B), the crystals presented as typical rhombohedra. No significant morphological change was found when rWLP was added at increasing concentrations (Fig 5C and 5D). Few crystals presented morphological changes after the induction by rWLP at high concentrations (50 μg/mL), and crystals with radial patterns were observed (Fig 5E and 5F). For the aragonite crystals, the rWLP showed significant effects on the crystal morphology. As shown in Fig 6, with increasing concentrations of rWLP, the native globular aragonite crystals changed from showing globular splitting to being peanut shaped.

**Fig 7:**
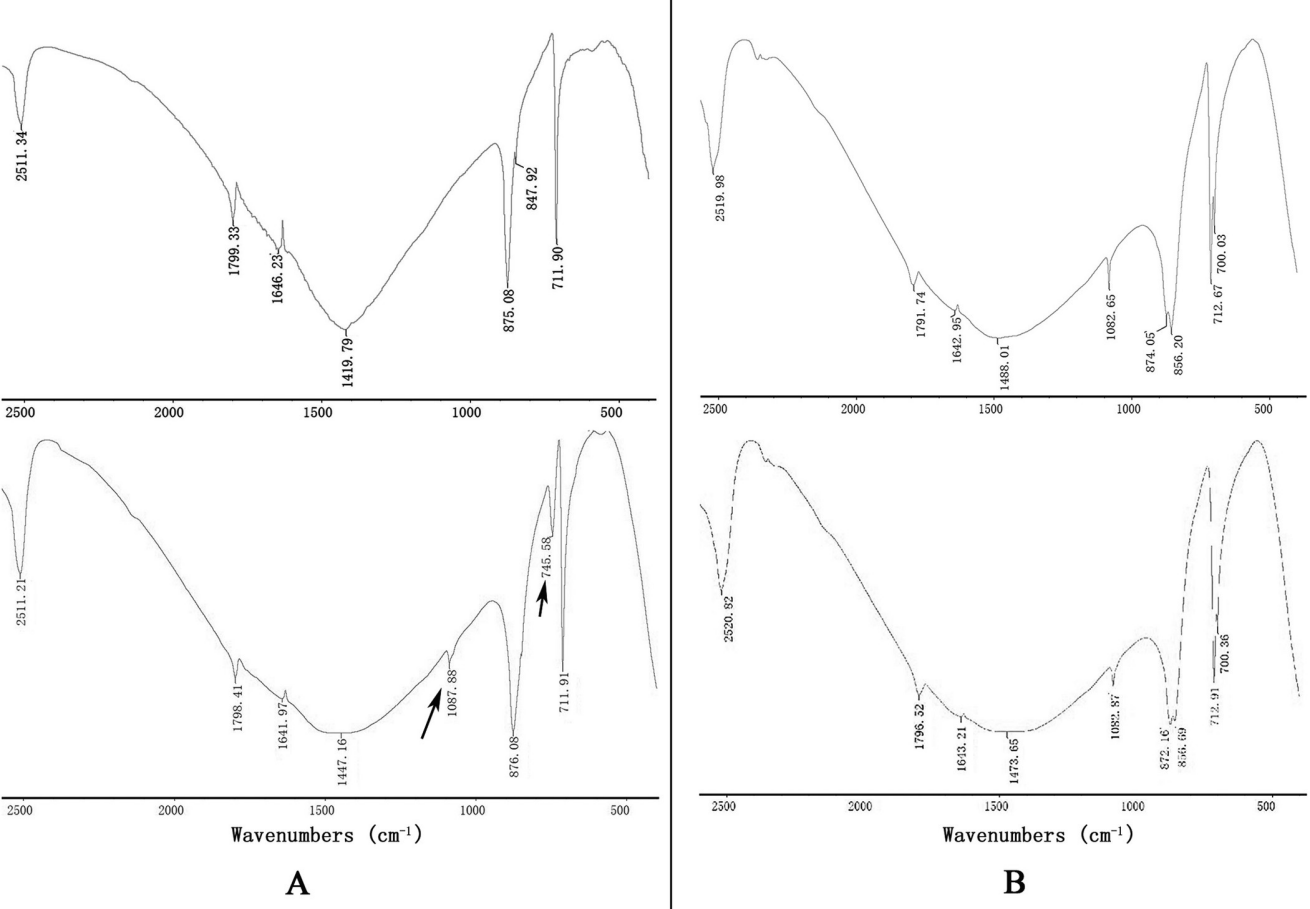
FTIR spectra of crystals induced by 50 μg/mL rWLP. A: the control (upper panel) and the rWLP induced (lower panel) calcite crystals; B: the control (upper panel) and the rWLP induced (lower panel) aragonite crystals. Arrows indicate the characteristic peaks of aragonite induced by rWLP.

FTIR was used to characterize the polymorphs of the induced crystals. As shown in Fig 7, the calcite crystals grown without protein induction were shown to be calcite with specific peaks (upper panel of Fig 7A). The rWLP-induced crystals showed extra aragonite-specific peaks with wavenumbers of 1087.88 and 745.58 (lower panel of Fig 7A). For the aragonite crystals, no changes in polymorphs were detected after rWLP was added to the solution (Fig 7B), indicating that rWLP had no effect on the polymorphs of the aragonite crystals.

**Fig 8:**
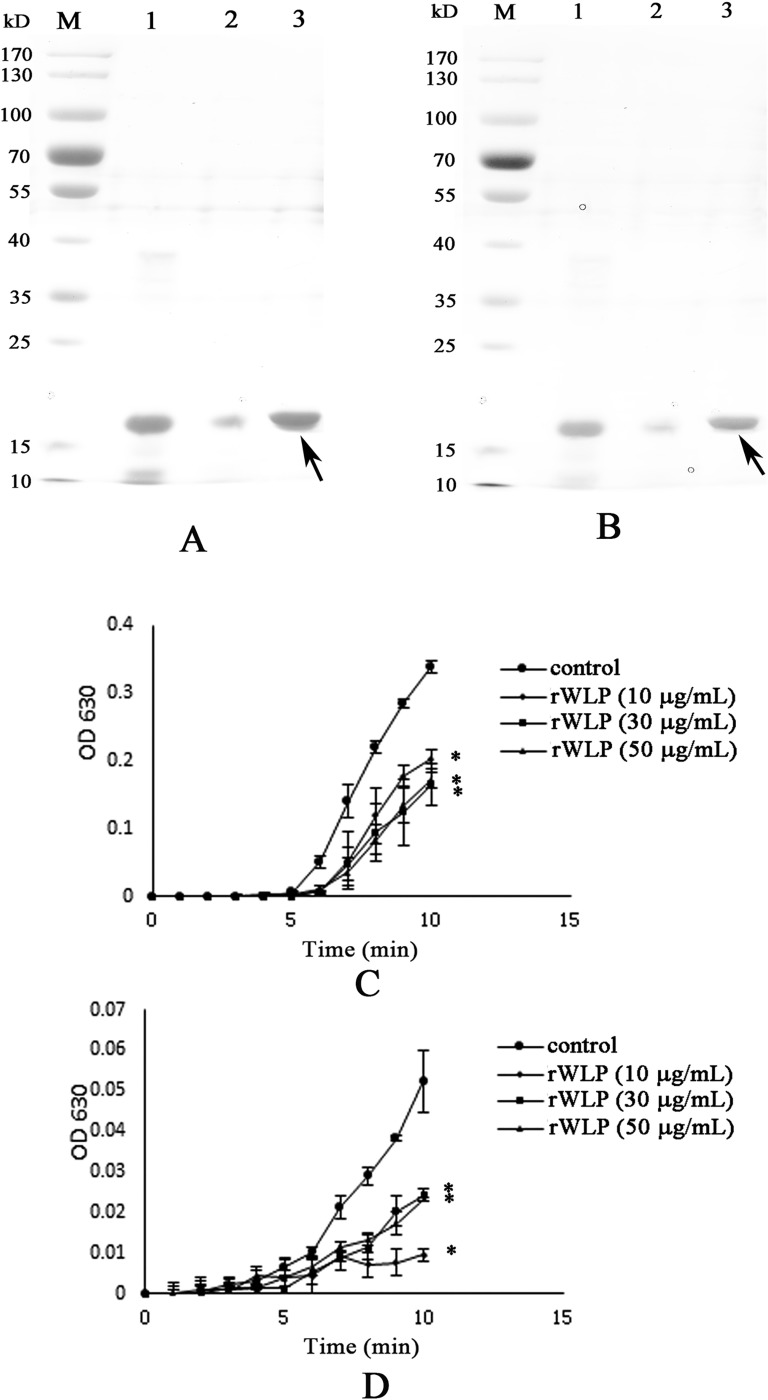
Binding ability of rWLP with calcite (A) and aragonite (B), and the crystallization rate inhibition of rWLP in calcite (C) and aragonite (D), respectively. Lane M, protein marker; Lane 1, pure rWLP; Lane 2, the supernatant of the solution after that the rWLP was precipitated by calcium carbonate crystals; Lane 3, the rWLP released from the precipitated calcium carbonate crystals; The Data of C and D represent mean ± SD (n = 3). *, p < 0.01.

SDS-PAGE was used to detect the possible interaction between rWLP and calcium carbonate crystals. As shown in Fig 8A and 8B, lanes 1, 2, and 3 present the pure rWLP solution, the supernatant of the rWLP solution after precipitation by calcium carbonate crystals, and the rWLP released from the precipitated calcium carbonate crystals, respectively. For both the calcite and aragonite crystals, the protein band of rWLP in lane 2 was much less intense than that in lane 1, revealing the binding of rWLP with the crystals, a finding that was confirmed by the reappearance of the protein band in lane 3 (Fig 8A and 8B).

The crystallization rate of calcium carbonate was measured by the absorbance at 630 nm. As shown in Fig 8C and 8D, rWLP significantly inhibited the crystallization rate of both the calcite and aragonite crystals, and the highest absorbance values, at 50 μg/mL of rWLP, were much lower than those of the control groups (0.2 *vs* 0.35 for calcite and 0.025 *vs* 0.055 for aragonite).

### Localization of WLP in the shell matrices and tissues of *M*. *coruscus*

**Fig 9:**
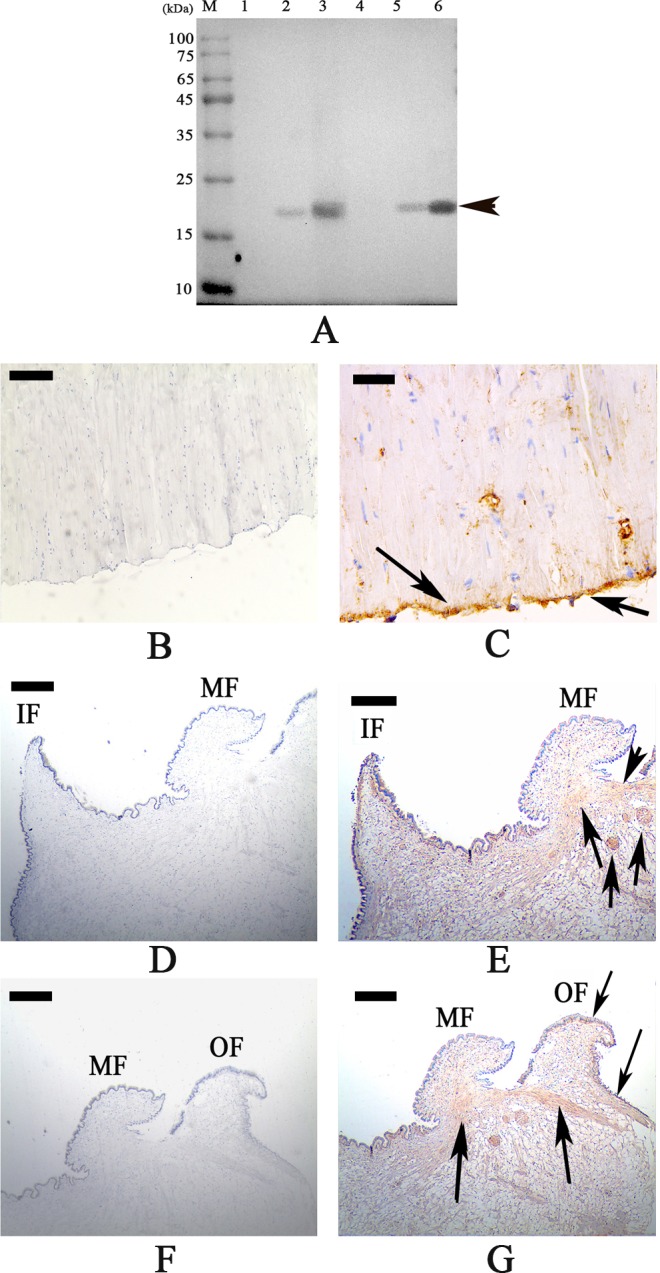
Western blot and immunohistochemistry analysis of WLP. A: Western blot by anti-rWLP antibody in shell matrices. Lane M, protein marker; Lane 1, soluble fraction from the fibrous prismatic layer; Lane 2, soluble fraction from the nacre layer; Lane 3, soluble fraction from the myostracum layer; Lane 4, insoluble fraction from the fibrous prismatic layer; Lane 5, insoluble fraction from the nacre layer; Lane 6, insoluble fraction from the myostracum layer. B: immunohistochemistry analysis with the control group of adductor muscle performed using only second antibody showed no significant signals; C: detection of WLP in the adductor muscle and the positive signals are indicated by arrows. D: the control group of mantle, showing the inner (IF) and the middle fold (MF) E: detection of WLP in the mantle and the positive signal is indicated by arrows; F: the control group of mantle, showing the middle (MF) and the outer fold (OF). G: detection of WLP in the mantle and the positive signals are indicated by arrows. The scale bar, 50 μm for A, C~F, and 25 μm for B.

Using the polyclonal anti-rWLP antibody prepared for this study, the presence of WLP in the three shell layers was investigated by western blotting. Shell proteins from the nacre, myostracum, and fibrous prism layer of *M*. *coruscus* were extracted and divided into two parts: acid-soluble and acid-insoluble. As shown in Fig 9A, WLP was detected with the expected MW in both the acid-soluble and acid-insoluble matrices from the myostracum and the nacre layer.

Immunohistochemistry analysis also revealed that the native WLP was expressed mainly at the bottom of the posterior adductor muscle, where it had a strong signal (Fig 9B and 9C). In addition, the expression of WLP in the mantle was detected with weak signal in the region under the outer fold and the middle fold, as well as at the edge of the outer fold (Fig 9D–9G).

### Identification of WLP protein partners in the shell matrices

Using Ni-coupled pull-down technology, rWLP with His_6_ tag was used as a bait, and the partner proteins in the shell matrices were pulled down. After LC-MS/MS analysis, a set of 10 proteins were identified with FDR<0.01 and matched unique peptides more than 2, including the WLP itself (Table 1). The MS/MS proteomics data have been deposited to the ProteomeXchange Consortium (http://proteomecentral.proteomexchange.org) via the iProX partner repository with the dataset identifier of PXD017074.

**Table 1:** LC-MS/MS identification of the protein partners of rWLP in the shell matrices extracted from the shell of *M*. *coruscus* by affinity adsorption using Ni-column pull down.

Protein IDs	Homologous name [species]	Homologous ID / E-value	Sequential features	Mascot Score	Sequence coverage	Matched Unique peptides	Intensity
Unigene68573	whirlin-like protein [*Mytilus coruscus*]	QGA67049.1 / 0.0	PDZ (SM000228)	323.3	100%	18	5.34E+11
CL1310.Contig2	PDZ domain-containing protein-1 [*Mytilus coruscus*]	AKS48171.1/ 0.0	Gln (22.5%); Pro (19.4%)	231.8	100%	11	6.23E+09
CL7444.Contig2	transgelin-like protein-3 [*Mytilus coruscus*]	AKS48154.1/ 4e-115	CH (SM000033)	140.5	97%	10	5.29E+10
CL7857.Contig1	shell mytilin-1 [*Mytilus coruscus*]	AKI87978.1/ 3e-130	Signal peptide (1–20); Leu (9.9%)	323.3	100%	9	1.05E+11
CL5847.Contig1	SD-rich protein-1 [*Mytilus coruscus*]	AKS48139.1/ 0.0	Internal repeat 1 Ser (17.8%); Asp (10.2%)	82.2	100%	8	2.57E+09
CL4409.Contig1	collagen-like protein-2 [*Mytilus coruscus*]	AKS48142.1 /0.0	VWA (SM000327)	66.4	100%	5	1.15E+10
CL700.Contig2	protease inhibitor-like protein-A [*Mytilus coruscus*]	AKS48173.1/ 2e-96	Signal peptide (1–22); NTR (SM000206)	19.1	89%	3	2.15E+08
Unigene69727	—	—	Gly (41.2%); Ser (14.7%); Asp (10.3%);	18.3	—	3	4.24E+08
CL6608.Contig1	—	—	Pro (32.5%); Ala (14.3%); Arg (13.0%);	12.8	—	2	1.99E+08
CL894.Contig1	actin [*Placida dendritica*]	ALU11283.1/ 6e-126	ACTIN(SM000268)	12.4	100%	2	7.12E+08

**Fig 10:**
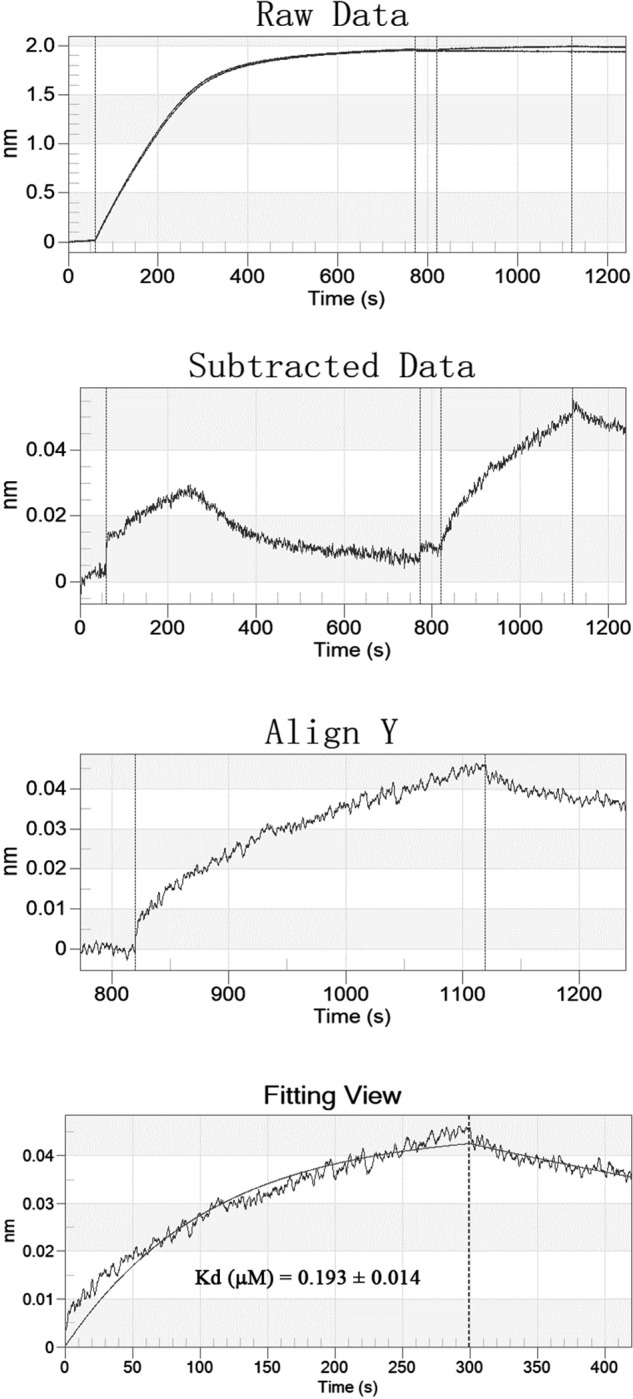
BLI curves (from the raw data to the final fitting view) for the binding of rWLP to biosensors coated with actin.

The binding of rWLP with actin was measured by BLI, and the raw data, subtracted data, aligned data, and final fitting are presented in Fig 10. The K_D_ of rWLP with actin was calculated as 0.193±0.014 μm (n = 3) by ForteBio data analysis software.

**Fig 11:**
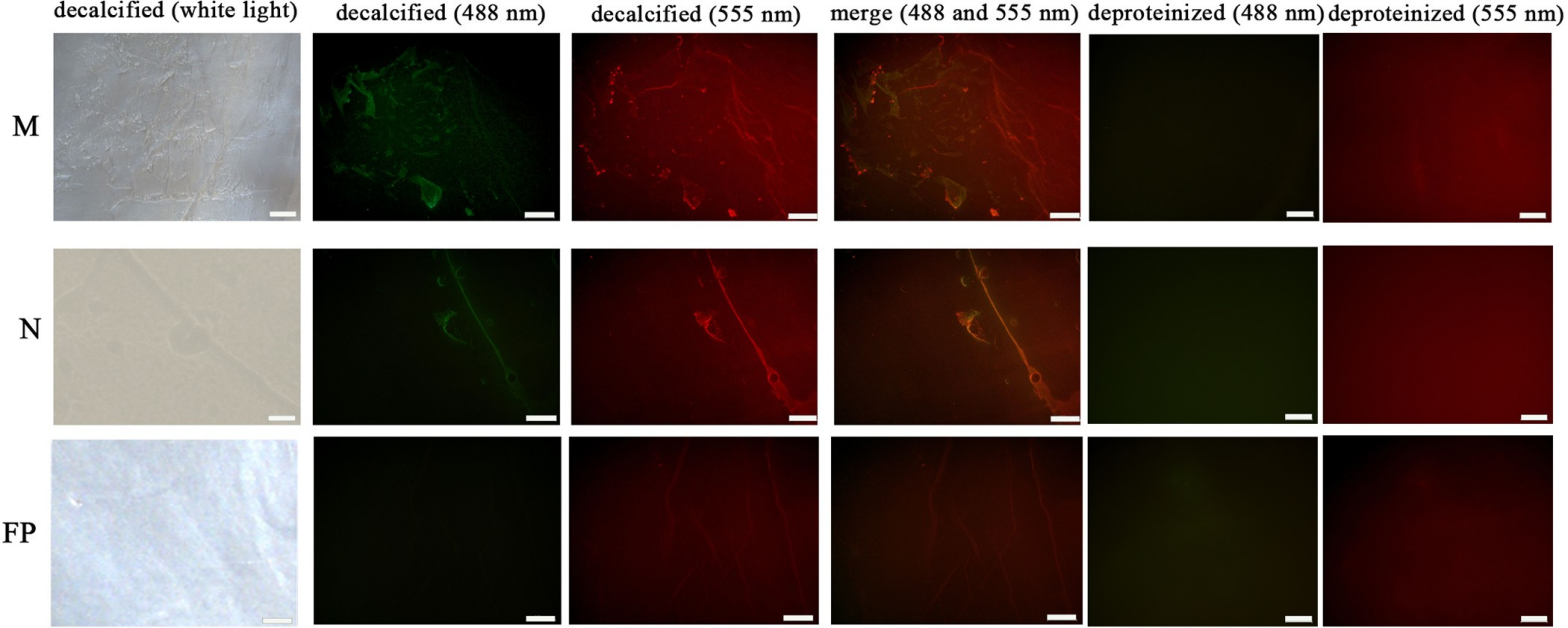
Immunofluorescence location of native WLP (green at 488 nm) and actin (red at 555 nm) on the surface of decalcified shell samples of *M*. *coruscus* and the deproteinized shell samples were used as negative controls. N, nacre; M, myostracum; FP, fibrous prism. The scale bar, 100 μm.

The microstructural location of WLP and actin on the shell surface was detected by double-labelling immunofluorescence (Fig 11). After decalcification, an organic membrane was presented on the shell surface with different textures of the myostracum, nacre, and fibrous prism layers (Fig 11). The signals of both WLP (green) and actin (red) were detected on the myostracum and the nacre layer of the decalcified shell surface. Most of the WLP and the actin signals were observed in the same region, suggesting interactions between these two proteins (Fig 11). No WLP signal was detected in the fibrous prismatic layer, but a weak actin signal was observed in this layer. For those deproteinated shell samples, no signal from WLP nor actin was detected (Fig 11).

## Discussion

SMPs have been reported as the predominant organic components that play crucial roles in *Mollusca* shell formation. More than 1 000 SMPs with very different structures have been identified in many mollusc shells, and most of these SMPs were identified through proteomic approaches. However, only a few SMPs have been characterized individually because of the extremely small amount of matrix proteins in the shell and the difficulty in obtaining enough protein samples for measurements. In this study, recombinant WLPs containing the PDZ domain were expressed successfully, and the function and location of these rWLPs were analysed to explore the possible mechanism of this protein in shell formation.

PDCPs have been previously identified in various mollusc shells, including WLP, PDZ/ZM, and PDZ/LIM domain-containing proteins [8, 23–26]. BLAST results revealed the high homology of WLP with PDCPs from other bivalves. In the Phylogenetic tree, two main clades were presented, the sequences from flatworm and the sequences from the shell-forming molluscs (also known as the conchiferans, including bivalves, scaphopods, gastropods and cephalopods). The clade of WLP (*M*. *coruscus*) was located between *Crassostrea* and *Mizuhopecten*, indicating the conservation of this protein among bivalves. As one type of PDCP, WLP has 147 residues, of which Gln is the most abundant amino acid (9.5%) in its sequence, followed by Gly (8.8%) and Arg (8.2%) (S2 Table). SMPs rich in Gln have been found in various *Mollusca* shells, such as those of *Pinctada margaritifera* [33], the gastropod *Lottia* [34], *Unionidae Elliptio complanata*, and *Villosa lienosa* [35]. Interestingly, vertebrate teeth also contain Gln-rich proteins, which are believed to interact with calcium ions and regulate tooth mineralization [36]. In parallel, it has been proposed that numerous Gln residues are implicated in the tensile mechanical properties of various silk-like fibroins [37]. In addition, WLP contains 39 (26.5%) charged amino acids (S2 Table). The charged amino acids of SMPs were proposed to play important roles in biomineralization, either by forming a "calcium bridge" with calcium ions or by interacting with negatively charged carbonate ions [29, 38–40]. The abundant Gln and charged amino acids of WLP indicate the function of this protein in biomineralization.

In this study, WLP was successfully expressed using a codon optimization strategy and a prokaryotic recombinant expression system. After isolation, renaturation, enterokinase digestion, and final purification, rWLP was collected, and the functions of rWLP were presented based on the results of *in vitro* calcium carbonate crystal induction, binding, and crystallization rate inhibition experiments. rWLP induced morphological changes in aragonite crystals rather than in calcite (Figs 5 and 6) and polymorphic changes in calcite crystals (Fig 7), exhibited binding with both calcite and aragonite crystals (Fig 8A and 8B), and inhibited the crystallization rate of both calcite and aragonite crystals (Fig 8C and 8D). As reported in previous works, SMPs can bind to the surface of calcium carbonate crystals, reduce the growth of crystals accordingly, and finally promote crystals formation with different morphologies and polymorphs [41]. The previous findings can partially explain the effects of rWLP on calcium carbonate crystallization but needs to be studied in more detail. We noted that the rWLP had relatively strong effects on the morphology of aragonite and the polymorph of calcite, suggesting the selective action of rWLP for different calcium carbonate crystals. In the shell of *M*. *coruscus*, the myostracum and the nacre are composed of aragonite with the same polymorph but different morphology, and WLP was identified exclusively in the myostracum layer [8]. Therefore, it is possible that the WLPs have important functions in the formation of the myostracum layer, such as the transformation of calcite to aragonite and the morphology change of aragonite.

Unlike most SMPs with the highest expression level in the mantle, the highest expression level of WLP was presented in adductor muscle and gonad (Fig 2A). Some SMP genes are expressed widely in multiple tissues [41, 42], suggesting that some SMPs may be produced by tissues other than the mantle. On the other hand, whirlin is an important protein family with multiple functions meditated by its PDZ domain [20]. The high expression level of WLP in adductor muscle, gonad, and blood cell suggested it may has fundamental roles beyond shell formation. Considering the important roles of the mantle in biomineralization, the highest expression level of WLP gene in the adductor muscle, and the possible roles of WLP in the attachment of shell-muscle, we performed *in situ* hybridization assays of WLP in these two tissues. For the mantle, the localization of the WLP gene in the epithelial cells of the middle fold and the outer fold (Fig 2D and 2E) implied a function of WLP in the formation of the prismatic layer [38–40]. Through the use of an anti-rWLP antibody, immunohistochemistry analysis confirmed the location of WLPs at the middle fold and the outer fold of the mantle (Fig 9). However, compared to the strong signal detected by *in situ* hybridization (Fig 2), the signal detected by immunohistochemistry was weak (Fig 9), suggesting a low abundance of native WLPs in the mantle. We speculate that most of the expressed WLPs may be transported from the mantle to the shell *via* the vesicular trafficking system, a principal pathway for the transportation of SMPs without signal peptides [41]. In addition, the expression level of the WLP gene was highest in the adductor muscle, which indicated the important function of WLP in this tissue. In *M*. *coruscus*, the adductor muscle is connected with the myostracum layer *via* organic membranes at the bottom of the adductor muscle and on the surface of the myostracum layer [8]. The strong WLP signal at the bottom of the adductor muscle, detected both by *in situ* hybridization (Fig 2) and immunohistochemistry (Fig 9), suggested the possible function of WLP in muscle-shell attachment.

As highlighted previously, the assembly of a biochemical framework is essential for shell formation [43]. In this study, 10 protein partners were identified by pull-down techniques combined with LC-MS/MS analyses (Table 1), providing a set of candidate proteins that interact with WLP. The main localization and function of these pulled-down proteins was the cytoskeleton (transgelin and actin), biomineralization (shell mytilin-1, PDZ domain-containing protein, and SD-rich protein), and others (collagen and protease inhibitor-like protein) (Table 1). Most of the pulled-down proteins had been identified in the shell proteome of *M*. *coruscus* [8], such as shell mytilin-1, a *Mytilus*-specific shell protein [8, 44], indicating a possible protein network in the shell mediated by WLP. On the other hand, most of the identified WLP protein partners are unsubstantiated, as are the functions of these interactions in shell formation. Among the identified WLP protein partners, actin was previously reported as a partner of PDCPs [45]. The BLI results in this study definitively showed the binding ability of rWLP with actin (Fig 10). Therefore, the location of WLP together with actin was further determined by double-labelling immunofluorescence. The results showed substantial signals of the two proteins and the overlapping pattern of WLP and actin on the shell surface of the myostracum layer (Fig 11). The identification of actin, and other cytoskeletal proteins in various mollusc shells has been controversial [8, 23, 26, 46]. We cannot exclude the possibility that actin may participate in biomineralization by interacting with actin-binding proteins (such as WLP in this study) and forming a framework during shell formation. Although more detailed studies are necessary for exploring the real interaction between WLP and the identified protein partners, our data provide clues for studying shell protein–protein interactions and bases for attempts at aiming to understand the supramolecular chemistry that contributes to shell formation.

In summary, WLP is a novel shell matrix protein with a PDZ domain, and the recombinant WLP expressed exhibited effects on the morphology, polymorphism, and crystallization rate of calcium carbonate crystals. The specific location of WLPs in the mantle, adductor muscle, and shell surface implied the functions of this protein in biomineralization and muscle-shell attachment. The protein partners of WLPs pulled down from shell matrices indicated a possible interaction framework in the shell, and the binding of WLPs with actin was verified. We are fully aware that the functional analysis of a single shell matrix protein is not enough to provide an explanation of the whole process of shell fabrication. However, we believe that characterization of biomineralization-related proteins will one-by-one reveal the complete biochemical framework required to precisely analyse the formation of mollusc shell.

## Supporting information

S1 Fig Alignment of WLP cDNA with the deduced amino acid sequence (GenBank QGA67049.1). The termination codon was denoted by an *.
(TIF)

S2 Fig Phylogenetic tree of WLP. The phylogenetic tree was constructed using MEGA 7.0 software with neighbor-joining method. Homologous included in construction of phylogenetic tree were retrieved from NCBI nr database with high score using BLAST. The BLAST information of selected sequences are shown in S1 Table.
(TIF)

S1 Table BLAST results of WLP in NCBI nr database. (DOCX)

S2 Table Amino acid composition (mole percent) of WLP. (DOCX)

S1 Raw images (PDF)
