# Supplementary figures and images for: Molecular characterization of a whirlin-like protein with biomineralization-related functions from the shell of Mytilus coruscus
Source: PLoS One. 2020 Apr 8;15(4):e0231414. doi: 10.1371/journal.pone.0231414 (PMC7141649; doi:10.1371/journal.pone.0231414)

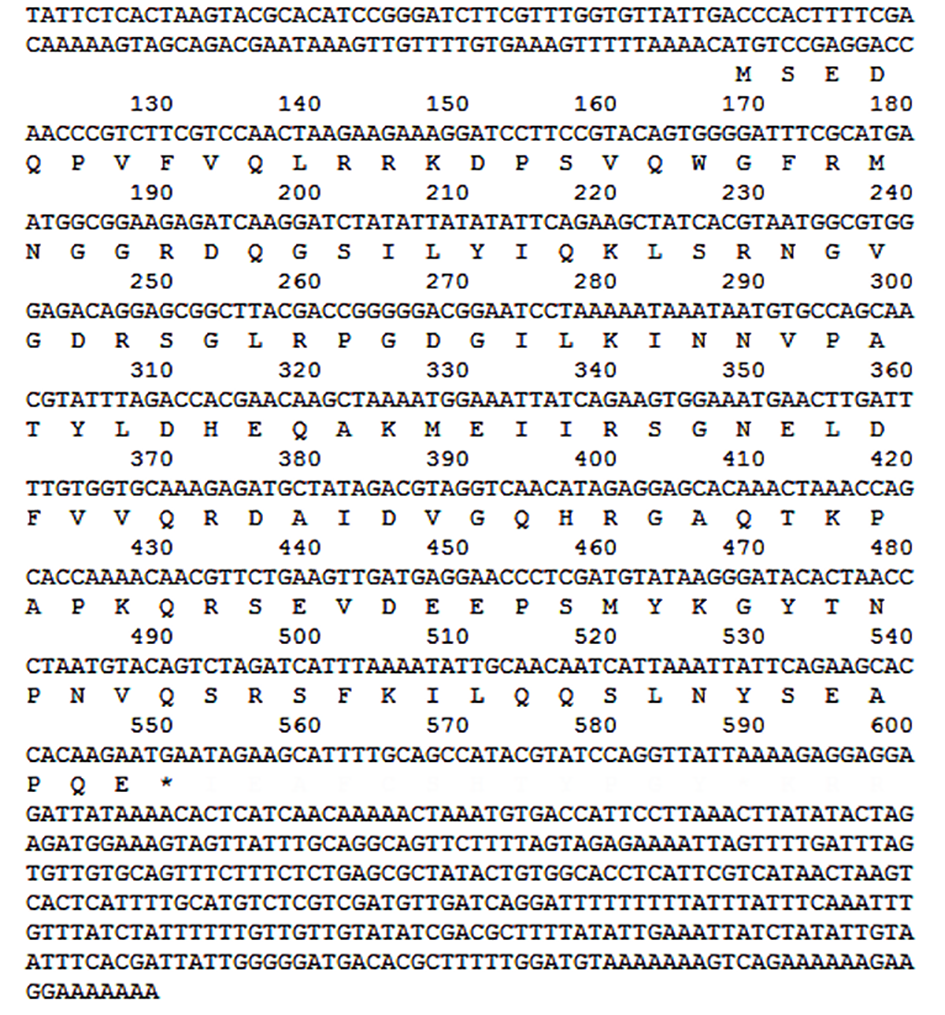

Supplement: S1 Fig — The termination codon was denoted by an *. (TIF) [file pone.0231414.s001.tif]

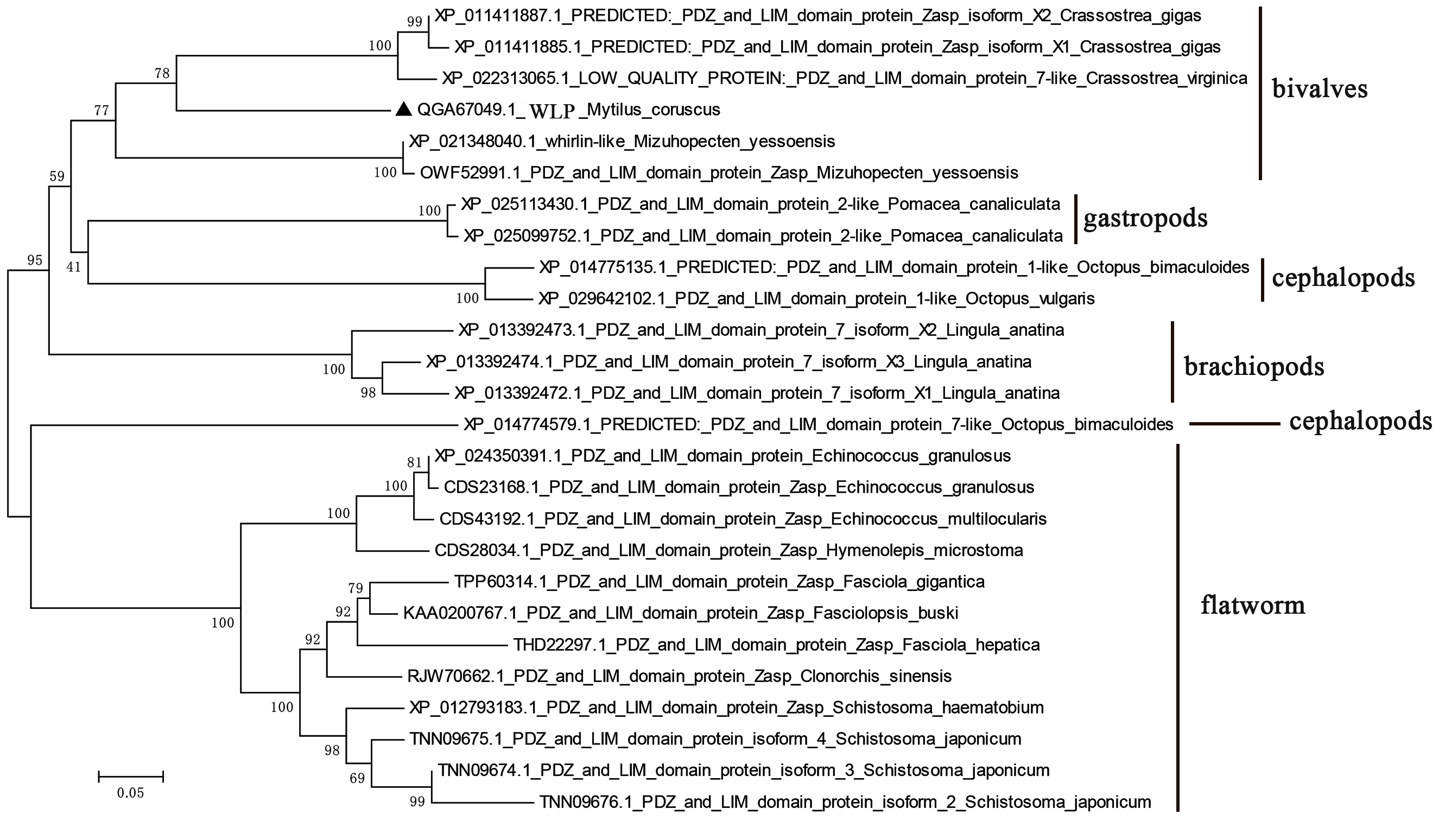

Supplement: S2 Fig — The phylogenetic tree was constructed using MEGA 7.0 software with neighbor-joining method. Homologous included in construction of phylogenetic tree were retrieved from NCBI nr database with high score using BLAST. The BLAST information of selected sequences are shown in S1 Table. (TIF) [file pone.0231414.s002.tif]
