# Supplementary material for: Molecular characterization of a whirlin-like protein with biomineralization-related functions from the shell of Mytilus coruscus
Source: PLoS One. 2020 Apr 8;15(4):e0231414. doi: 10.1371/journal.pone.0231414 (PMC7141649; doi:10.1371/journal.pone.0231414)
Supplement: S1 Table — (DOCX) [file pone.0231414.s003.docx]

| **Description** | **Species** | **Max Score** | **Total Score** | **Query Cover** | **E value** | **Per. Ident** | **Accession** |
| --- | --- | --- | --- | --- | --- | --- | --- |
| LOW QUALITY PROTEIN: PDZ and LIM domain protein 7-like | Crassostrea virginica | 183 | 183 | 96% | 2.00E-56 | 62.33% | XP_022313065.1 |
| PREDICTED: PDZ and LIM domain protein Zasp isoform X2 | Crassostrea gigas | 179 | 179 | 97% | 3.00E-54 | 60.14% | XP_011411887.1 |
| PREDICTED: PDZ and LIM domain protein Zasp isoform X1 | Crassostrea gigas | 179 | 179 | 95% | 6.00E-54 | 61.38% | XP_011411885.1 |
| whirlin-like | Mizuhopecten yessoensis | 145 | 145 | 93% | 9.00E-42 | 51.82% | XP_021348040.1 |
| PDZ and LIM domain protein Zasp | Mizuhopecten yessoensis | 145 | 145 | 93% | 1.00E-41 | 51.82% | OWF52991.1 |
| PDZ and LIM domain protein 2-like | Pomacea canaliculata | 127 | 127 | 94% | 8.00E-35 | 43.88% | XP_025113430.1 |
| PDZ and LIM domain protein 2-like | Pomacea canaliculata | 127 | 127 | 94% | 9.00E-35 | 43.88% | XP_025099752.1 |
| PREDICTED: PDZ and LIM domain protein 1-like | Octopus bimaculoides | 118 | 118 | 89% | 2.00E-30 | 46.21% | XP_014775135.1 |
| PDZ and LIM domain protein Zasp | Hymenolepis microstoma | 117 | 117 | 94% | 3.00E-30 | 41.45% | CDS28034.1 |
| PDZ and LIM domain protein 1-like | Octopus vulgaris | 115 | 115 | 89% | 4.00E-29 | 44.70% | XP_029642102.1 |
| PDZ and LIM domain protein 7 isoform X3 | Lingula anatina | 113 | 113 | 97% | 7.00E-29 | 40.25% | XP_013392474.1 |
| PDZ and LIM domain protein | Echinococcus granulosus | 112 | 112 | 94% | 4.00E-28 | 40.13% | XP_024350391.1 |
| PDZ and LIM domain protein 7 isoform X1 | Lingula anatina | 111 | 111 | 91% | 5.00E-28 | 41.72% | XP_013392472.1 |
| PDZ and LIM domain protein Zasp | Clonorchis sinensis | 112 | 112 | 93% | 6.00E-28 | 41.06% | RJW70662.1 |
| PDZ and LIM domain protein Zasp | Fasciola gigantica | 110 | 110 | 91% | 1.00E-27 | 39.86% | TPP60314.1 |
| PDZ and LIM domain protein Zasp | Schistosoma haematobium | 110 | 110 | 91% | 2.00E-27 | 41.22% | XP_012793183.1 |
| PDZ and LIM domain protein Zasp | Echinococcus multilocularis | 112 | 112 | 94% | 3.00E-27 | 40.13% | CDS43192.1 |
| PDZ and LIM domain protein isoform 3 | Schistosoma japonicum | 109 | 109 | 91% | 4.00E-27 | 39.19% | TNN09674.1 |
| PDZ and LIM domain protein Zasp | Echinococcus granulosus | 112 | 112 | 94% | 4.00E-27 | 40.13% | CDS23168.1 |
| PDZ and LIM domain protein isoform 4 | Schistosoma japonicum | 109 | 109 | 91% | 7.00E-27 | 39.19% | TNN09675.1 |
| PDZ and LIM domain protein isoform 2 | Schistosoma japonicum | 109 | 109 | 91% | 7.00E-27 | 39.19% | TNN09676.1 |
| PDZ and LIM domain protein Zasp | Fasciolopsis buski | 108 | 108 | 91% | 1.00E-26 | 39.19% | KAA0200767.1 |
| PDZ and LIM domain protein 7 isoform X2 | Lingula anatina | 105 | 105 | 97% | 1.00E-25 | 37.11% | XP_013392473.1 |
| PREDICTED: PDZ and LIM domain protein 7-like | Octopus bimaculoides | 100 | 100 | 87% | 1.00E-23 | 38.93% | XP_014774579.1 |
| PDZ and LIM domain protein Zasp | Fasciola hepatica | 100 | 100 | 91% | 2.00E-23 | 34.90% | THD22297.1 |
| PDZ and LIM domain protein 5-like | Pomacea canaliculata | 97.8 | 97.8 | 90% | 2.00E-22 | 38.97% | XP_025082862.1 |
| PREDICTED: PDZ and LIM domain protein 7-like | Aplysia californica | 94.7 | 94.7 | 99% | 4.00E-21 | 35.48% | XP_005103005.2 |
| PDZ and LIM domain protein 3-like isoform X2 | Pomacea canaliculata | 97.1 | 97.1 | 58% | 5.00E-21 | 50.00% | XP_025114462.1 |
| PREDICTED: PDZ and LIM domain protein 7-like | Biomphalaria glabrata | 94.4 | 94.4 | 98% | 6.00E-21 | 34.67% | XP_013091711.1 |
| PDZ and LIM domain protein 3-like isoform X1 | Pomacea canaliculata | 96.7 | 96.7 | 58% | 9.00E-21 | 50.00% | XP_025114384.1 |
| PREDICTED: PDZ and LIM domain protein 3 isoform X2 | Crassostrea gigas | 95.9 | 95.9 | 58% | 1.00E-20 | 54.65% | XP_011437719.1 |
| PREDICTED: PDZ and LIM domain protein 1 isoform X1 | Crassostrea gigas | 96.3 | 96.3 | 58% | 2.00E-20 | 54.65% | XP_011437718.1 |
| PDZ and LIM domain protein 3-like isoform X2 | Crassostrea virginica | 94.7 | 94.7 | 58% | 4.00E-20 | 54.65% | XP_022313541.1 |
| PDZ and LIM domain protein 3-like isoform X1 | Crassostrea virginica | 94.7 | 94.7 | 59% | 6.00E-20 | 53.41% | XP_022313540.1 |
| PREDICTED: PDZ and LIM domain protein 3-like isoform X2 | Octopus bimaculoides | 93.2 | 93.2 | 58% | 7.00E-20 | 46.51% | XP_014769275.1 |
| PREDICTED: PDZ and LIM domain protein 1-like isoform X1 | Octopus bimaculoides | 93.6 | 93.6 | 58% | 9.00E-20 | 46.51% | XP_014769274.1 |
| PREDICTED: PDZ and LIM domain protein 3-like isoform X3 | Octopus bimaculoides | 92.4 | 92.4 | 58% | 1.00E-19 | 46.51% | XP_014769276.1 |
| PDZ and LIM domain protein Zasp-like isoform X3 | Octopus vulgaris | 92.4 | 92.4 | 58% | 2.00E-19 | 46.51% | XP_029639600.1 |
| PDZ and LIM domain protein 3-like isoform X2 | Octopus vulgaris | 92.8 | 92.8 | 58% | 2.00E-19 | 46.51% | XP_029639599.1 |
| PDZ and LIM domain protein 3-like isoform X1 | Octopus vulgaris | 93.2 | 93.2 | 58% | 2.00E-19 | 46.51% | XP_029639598.1 |
| PDZ and LIM domain protein 3 isoform X2 | Lingula anatina | 92.8 | 92.8 | 70% | 6.00E-19 | 38.66% | XP_013392477.1 |
| PDZ and LIM domain protein 3 isoform X1 | Lingula anatina | 92.8 | 92.8 | 70% | 6.00E-19 | 38.66% | XP_013392476.1 |
| PDZ and LIM domain protein Zasp | Hymenolepis microstoma | 90.1 | 90.1 | 57% | 7.00E-19 | 47.06% | CDS32949.1 |
| PDZ and LIM domain protein 3 | Clonorchis sinensis | 90.5 | 90.5 | 68% | 1.00E-18 | 42.59% | RJW73901.1 |
| PDZ and LIM domain protein Zasp-like | Crassostrea virginica | 87 | 87 | 97% | 1.00E-18 | 36.81% | XP_022337598.1 |
| PDZ and LIM domain protein Zasp | Hymenolepis microstoma | 89.4 | 89.4 | 57% | 2.00E-18 | 50.59% | CDS32950.1 |
| PDZ and LIM domain protein Zasp | Mizuhopecten yessoensis | 87.8 | 87.8 | 63% | 6.00E-18 | 44.68% | OWF46097.1 |
| PDZ and LIM domain protein 1-like isoform X2 | Mizuhopecten yessoensis | 88.6 | 88.6 | 63% | 7.00E-18 | 44.68% | XP_021362430.1 |
| PREDICTED: PDZ and LIM domain protein Zasp-like | Crassostrea gigas | 85.1 | 85.1 | 97% | 7.00E-18 | 35.42% | XP_011413342.1 |
| PDZ and LIM domain protein | Echinococcus granulosus | 87.8 | 87.8 | 58% | 8.00E-18 | 51.16% | XP_024353600.1 |
| PREDICTED: PDZ and LIM domain protein 3-like isoform X3 | Aplysia californica | 88.2 | 88.2 | 58% | 9.00E-18 | 46.51% | XP_012944396.1 |
| PREDICTED: PDZ and LIM domain protein 3-like isoform X2 | Aplysia californica | 88.2 | 88.2 | 58% | 1.00E-17 | 46.51% | XP_012944395.1 |
