# Supplementary material for: Molecular characterization of a whirlin-like protein with biomineralization-related functions from the shell of Mytilus coruscus
Source: PLoS One. 2020 Apr 8;15(4):e0231414. doi: 10.1371/journal.pone.0231414 (PMC7141649; doi:10.1371/journal.pone.0231414)
Supplement: S2 Table — (DOCX) [file pone.0231414.s004.docx]

**S2 Table**

| Amino acid | Mole percent |
| --- | --- |
| Ala (A) | 4.10% |
| Arg (R) | 8.20% |
| Asn (N) | 5.40% |
| Asp (D) | 6.80% |
| Cys (C) | 0.00% |
| Gln (Q) | 9.50% |
| Glu (E) | 6.10% |
| Gly (G) | 8.80% |
| His (H) | 1.40% |
| Ile (I) | 5.40% |
| Leu (L) | 6.10% |
| Lys (K) | 5.40% |
| Met (M) | 2.70% |
| Phe (F) | 2.70% |
| Pro (P) | 6.10% |
| Ser (S) | 8.20% |
| Thr (T) | 2.00% |
| Trp (W) | 0.70% |
| Tyr (Y) | 3.40% |
| Val (V) | 6.80% |
| Pyl (O) | 0.00% |
| Sec (U) | 0.00% |
