# Supplementary material for: Molecular characterization of a whirlin-like protein with biomineralization-related functions from the shell of Mytilus coruscus
Source: PLoS One. 2020 Apr 8;15(4):e0231414. doi: 10.1371/journal.pone.0231414 (PMC7141649; doi:10.1371/journal.pone.0231414)
Supplement: S1 Raw images — (PDF) [file pone.0231414.s005.pdf]

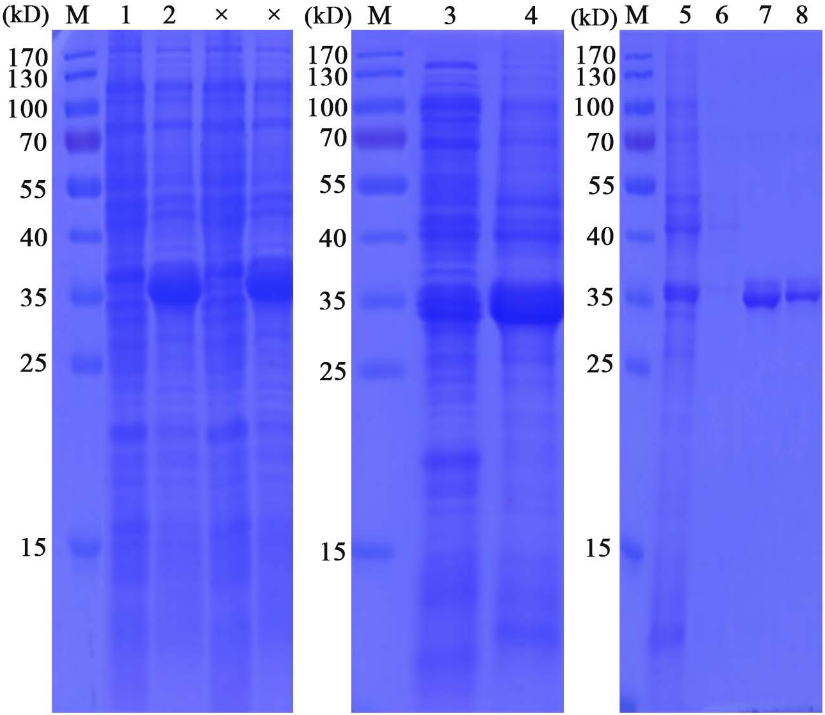

Original images for Fig.3. Expression and isolation of rWLP.

Lane M, Protein Marker;

Lane 1, negative control without the IPTG induction;

Lane 2, expression of rWLP with the induction of IPTG;

Lane 3, the supernatant of cell lysate;

Lane 4 and 5, the debris of cell lysate;

Lane 6, eluted rWLP from Ni-NTA column with 30 mM imidazole.

Lane 7, eluted rWLP from Ni-NTA column with 300 mM imidazole;

Lane 8, eluted rWLP from Ni-NTA column with 500 mM imidazole.

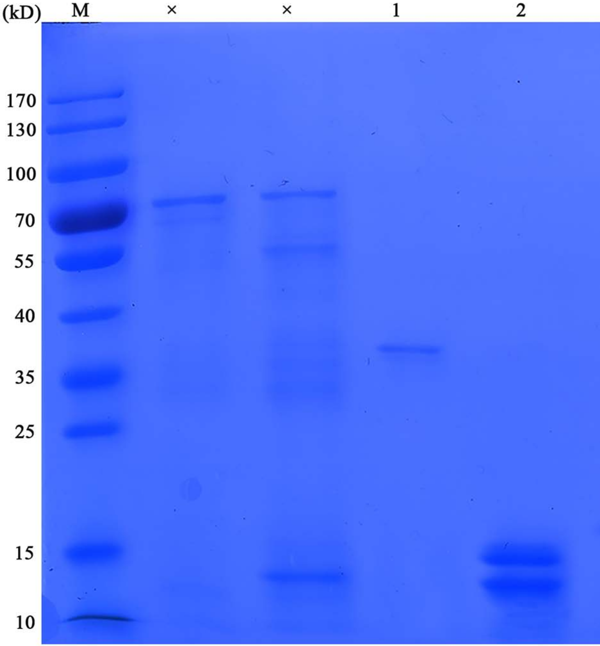

Original images for Fig.4A. Enterokinase digestion of rWLP

Lane M, Protein Marker;

lane 1, rWLP before enterokinase digestion;

lane 2, rWLP after enterokinase digestion.

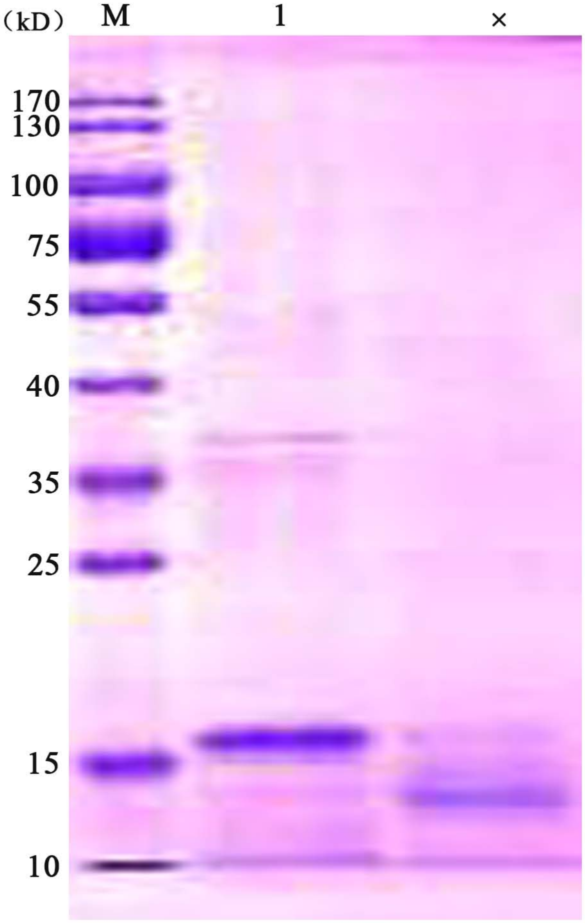

Original images for Fig.4B.

SDS-PAGE of the purified rWLP after enterokinase digestion

Lane M, Protein Marker;

lane 1, rWLP after enterokinase digestion and HPLC purification

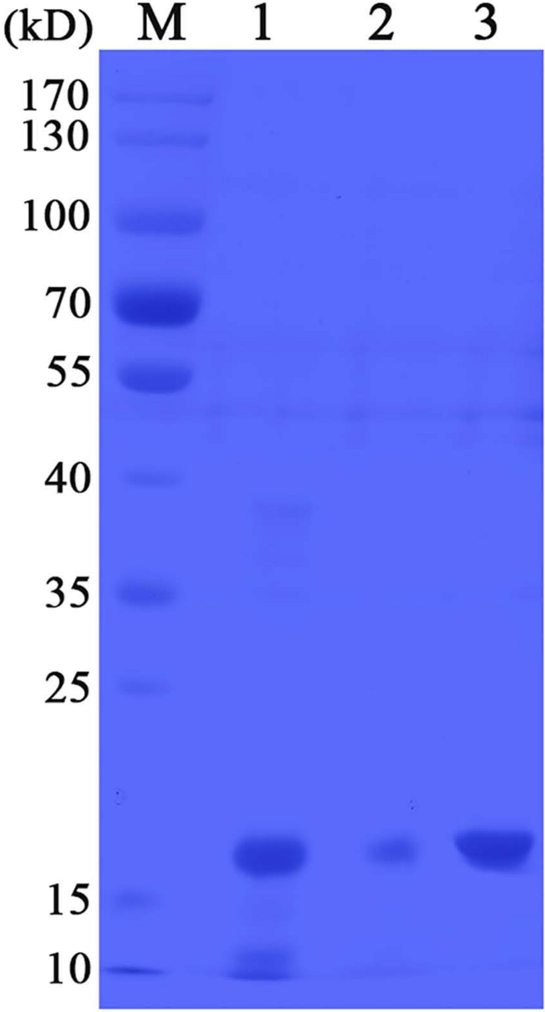

Original image for Fig.8A.

Binding ability of rWLP with calcite

Lane M, protein marker;

lane 1, pure rWLP;

Lane 2, the supernatant of the solution after that the rWLP was precipitated by calcite;

Lane 3, the rWLP released from the precipitated calcite

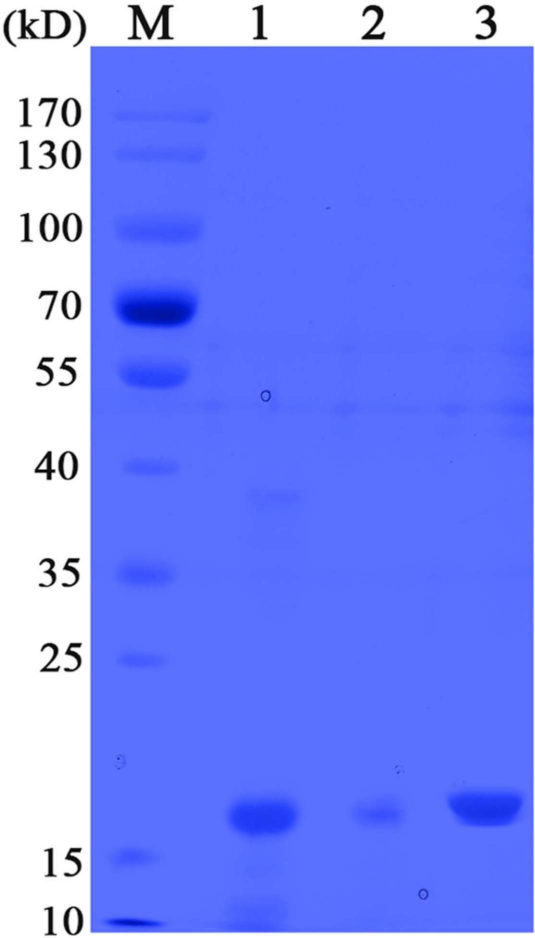

Original image for Fig.8B.

Binding ability of rWLP with aragonite

Lane M, protein marker;

lane 1, pure rWLP;

Lane 2, the supernatant of the solution after that the rWLP was precipitated by aragonite;

Lane 3, the rWLP released from the precipitated aragonite

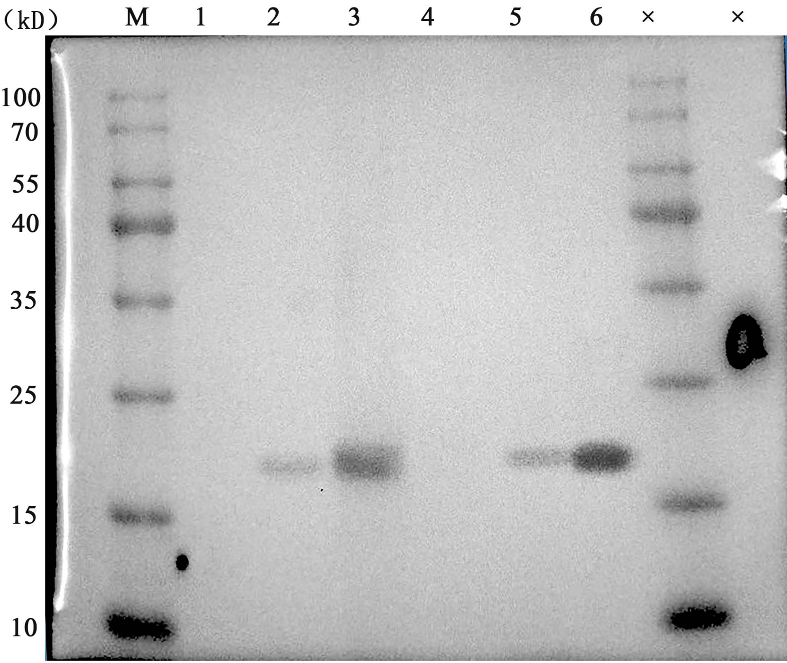

Original image for Fig.9A.

Western blot by anti-rWLP antibody in shell matrices.

Lane M, protein marker;

Lane 1, soluble fraction from the fibrous prismatic layer;

Lane 2, soluble fraction from the nacre layer;

Lane 3, soluble fraction from the myostracum layer;

Lane 4, insoluble fraction from the fibrous prismatic layer;

Lane 5, insoluble fraction from the nacre layer;

Lane 6, insoluble fraction from the myostracum layer.
